# A Multiobjective, Lion Mating Optimization Inspired Routing Protocol for Wireless Body Area Sensor Network Based Healthcare Applications

**DOI:** 10.3390/s19235072

**Published:** 2019-11-20

**Authors:** Muhammad Faheem, Rizwan Aslam Butt, Basit Raza, Hani Alquhayz, Muhammad Zahid Abbas, Md Asri Ngadi, Vehbi Cagri Gungor

**Affiliations:** 1 Department of Computer Science, Universiti Teknologi Malaysia, Johor Bahru 81310, Malaysia; 2 Department of Computer Engineering, Abdullah Gul University, Kayseri 38080, Turkey; cagri.gungor@agu.edu.tr; 3 Department of Electronics Engineering, NED University of Engineering, Karachi 75270, Pakistan; rizwan.aslam@neduet.edu.pk; 4 Department of Computer Science, COMSATS University Islamabad (CUI), Islamabad 45550, Pakistan; basit.raza@comsats.edu.pk; 5 Department of Computer Science and Information, College of Science in Zulfi, Majmaah University, Al-Majmaah 11952, Saudi Arabia; h.alquhayz@mu.edu.sa; 6 Department of Computer Science, COMSATS University, Vehari Campus 61100, Pakistan; zahidabbas@ciitvehari.edu.pk

**Keywords:** Internet of things, body area network, body area sensor network, biomedical sensors, healthcare, routing protocol

## Abstract

The importance of body area sensor networks (BASNs) is increasing day by day because of their increasing use in Internet of things (IoT)-enabled healthcare application services. They help humans in improving their quality of life by continuously monitoring various vital signs through biosensors strategically placed on the human body. However, BASNs face serious challenges, in terms of the short life span of their batteries and unreliable data transmission, because of the highly unstable and unpredictable channel conditions of tiny biosensors located on the human body. These factors may result in poor data gathering quality in BASNs. Therefore, a more reliable data transmission mechanism is greatly needed in order to gather quality data in BASN-based healthcare applications. Therefore, this study proposes a novel, multiobjective, lion mating optimization inspired routing protocol, called self-organizing multiobjective routing protocol (SARP), for BASN-based IoT healthcare applications. The proposed routing scheme significantly reduces local search problems and finds the best dynamic cluster-based routing solutions between the source and destination in BASNs. Thus, it significantly improves the overall packet delivery rate, residual energy, and throughput with reduced latency and packet error rates in BASNs. Extensive simulation results validate the performance of our proposed SARP scheme against the existing routing protocols in terms of the packet delivery ratio, latency, packet error rate, throughput, and energy efficiency for BASN-based health monitoring applications.

## 1. Introduction

The ratio of the aged population is increasing globally. Aged persons are at higher risk of diseases. Especially, there are some chronic, life-threatening diseases such as diabetes and cardiac arrest that require continuous monitoring of patients. On the other hand, healthcare expenditures have been growing enormously, which is an extremely heavy burden on the country’s economy [1,2]. At the same time, there is a huge proportion of people that receive inadequate healthcare monitoring because of scarce medical resources compared to the great volume of demands [3]. There are currently 180 million people worldwide that suffer from diabetes, and the number is expected to grow to 360 million by 2030 [4]. Debilitating neurodegenerative disease such as Parkinson’s affects even more. These patients’ lives can be greatly relieved if a low-cost health monitoring system is established to provide continuous services to the public. This will also help to relieve the growing pressure imposed on public healthcare expenditure. As indicated by recent research, body area sensor networks (BASNs) are a promising choice for building such health monitoring systems [5]. BASNs offer new opportunities for health monitoring applications such as the quick setting up of a communication network without any infrastructure support in a self-organizing manner to provide robust, continuous, and reliable communication in dynamic situations. There are two basic subcategories of the BASNs in medical applications, namely, implant BASNs and wearable BASNs for remote monitoring and control of the patients [6]. A wearable BASN is capable of monitoring sleep disorders, asthma, battle readiness, and many other human body conditions. For implant BASNs, the biosensors are either implanted below the surface skin or reside in the bloodstream. Functions such as diabetes control, cardiovascular disease monitoring, and cancer detection can be fulfilled by implanting BASNs. The most important uses of such networks are to measure different physiological markers of the person under testing (e.g., temperature, electrocardiogram (ECG), blood oxygen level), control the environment surrounding his body, track his motion behavior, and, subsequently send this data to a control center for real-time observations. Therefore, the medical care system is one of the most crucial application scenarios for BASNs [7,8]. In BASNs, each biosensor consists of a sensing unit, power unit, communication unit, storage unit, and a processing unit. These tiny medical sensors are located on the surface of, or implanted inside, the human body to monitor the health status, and they send the captured bio-signals wirelessly to a data center connected to a back-end medical system that analyses these signals for diagnostic analysis [9]. 

Typical architecture of a BASN employs a coordinator node to communicate with the body sensors and aggregate the data from all the biosensors. The coordinator then sends the collected data to the remote server through a cellular network, WiFi, or any other long-distance communication system. Therefore, IoT-enabled BASN technologies have emerged as a promising networking paradigm for e-healthcare systems [10,11]. Consequently, with the instant data collected from body sensors, healthcare providers such as doctors and nurses will have a much better picture of the health condition of their patients [12]. Thus, doctors can remotely obtain all the information needed from the wireless sensors worn by the patient and the historical data stored in the database to identify primary symptoms of a given disease and detect health degradation. Thus, the gathered data significantly help to better anticipate risks, manage rescue operations, reduce mistakes, and assist in life-critical operations. This health status monitoring system allows fast reactions to health problems for a wide range of populations, regardless of where these people are located. Consequently, it facilitates and minimizes the workload of doctors in clinical centers by allowing continuous remote patient monitoring. Since the biosensors are low-cost, compared to the expensive and secure equipment in the hospital, a much larger population can be covered by the BASN monitoring system than the traditional medical care system. Thus, it frees patients from intensive personal care [13]. However, the wide applications of BASNs in the medical setting are seriously hindered by concerns such as reliable data transmission and the short life span of the embedded batteries of the sensor units. Though wireless communication in BASN is limited to an area of several meters wide, the channel conditions on the human body are highly unstable and unpredictable, which imposes great challenges for reliable data transmission [14,15]. Therefore, an effective data transmission strategy needs to be developed to enhance data reliability with reduced power consumption of the biosensor nodes as much as possible in the network. Therefore, in this study, a novel, multiobjective, lion mating optimization inspired routing protocol for BASN-based IoT health monitoring applications is presented. 

In the rest of the paper, Section 2 discusses the existing routing protocols in BASNs. In Section 3, we briefly describe the research challenges and research motivations. In Section 4, we discuss our proposed SARP routing protocol. In Section 5, we discuss the channel and energy consumption models used in the simulation studies. In Section 6, we provide simulation results and analyze the performance of SARP against existing routing protocols in BASNs. Finally, in Section 7, we conclude the paper with future works.

## 2. Existing Studies in BASNs

The traffic generated from BASNs can be classified into two main classes, namely, real-time traffic (having stringent temporal requirements) and non-real-time traffic (that tolerates delays and requires reliable delivery). In the literature, some research efforts have been made to improve communication reliability with low energy consumption in BASNs. For example, the work in [16] proposes a cooperative routing protocol to increase the overall packet delivery ratio and throughput in BASNs. The proposed scheme employs a linear, three-node arrangement with an amplifying-and-forwarding mechanism to route packets over the shortest path towards the sink. The proposed protocol improves network throughput at the cost of high latency and packet error rates. The study in [17] presents a routing protocol that minimizes latency and energy consumption issues in BASNs. The proposed scheme also routes data packets over the shortest path from the source towards the sink. This mechanism may be helpful to minimize the latency but leads to the issue of network congestion, which increases packet losses or invalid data packets in BASNs. Likewise, the authors in [18] propose a routing protocol to distribute the energy consumption load evenly among the biosensors in order to extend the lifetime of the BASNs. The proposed scheme shows a significant improvement in terms of throughput, residual energy, and network lifetime. However, because poor synchronization between the biosensors leads to excessive interference, there is a large number of corrupted data packets. Hence, the overall effective packet delivery ratio decreases in BASNs. 

Similarly, an optimized routing approach in [19] is proposed to enhance the routing performance of data gathering for critical and emergency networks. The developed beacon-less protocol relies on advertisement packets to establish reliable routing paths between the source and destination in the network. The proposed mechanism significantly reduces the latency as a trade-off with the poor network scalability. The work in [20] isolates the offending nodes in order to provide reliable data delivery in BASNs. The proposed trust and thermal-aware routing protocol, because it employs a multifaceted routing strategy, finds the best relay nodes to cope with hotspot and load balancing issues. The developed protocol decreases the packet drop ratio and latency and increases the packet delivery ratio with high network throughput in BASNs. The researchers in [21] propose a communication framework to relay data packets over highly reliable links towards the sink in BASNs. In the proposed scheme, the authors presume that the nodes exhibiting periodic channel fluctuations can be divided into various groups: nodes on the right side of the body and nodes on the left. However, poor synchronization between biosensors is another issue, which also results in significant packet loss, particularly for the biosensors closer to the sink. In [12], an efficient and reliable packet transmission mechanism is proposed, which takes advantage of periodic human movements such as walking and running. The proposed scheme achieves high packet delivery rates at a lower energy consumption, but it faces synchronization issues and latency with packet arrivals. Also, a thermal-aware packet forwarding scheme is proposed in [22] to handle temperature rise issues in BASNs. The suggested scheme employs a multiring-like packet forwarding architecture to find alternative routing paths towards the sink. 

The work in [2] also presents a data priority-aware routing protocol to maximize packet delivery rates with low energy consumption and latency in BASNs. The network clustering approaches have proven to be an effective solution for organizing energy-constrained biosensor networks by minimizing energy consumption and reducing communication costs. Clustering techniques aim at gathering data among groups of elementary biosensor nodes, which elect their leaders (the cluster, head node) among themselves. The cluster leader is mainly a powerful node compared to elementary sensors that are in charge of performing data aggregation and communication to the terminal base station. Inspired by the above-mentioned ideas, the work in [23] discusses a cluster-based routing protocol for BASNs. In the proposed protocol, the biosensors are partitioned into groups called clusters. Each cluster has several member nodes, and the main coordinator (referred to as a cluster head) is responsible for collecting periodic or streamed data about the phenomenon of interest from the member nodes. The cluster heads aggregate the collected data and send it directly to the terminal base station or they use multihop communications. Similarly, the study in [24] proposes a dual sink clustering routing protocol for reliable packet transmission in BASNs. However, the use of a dual sink increases the overall network deployment cost and network complexity. Also, the ratio of the number of incorrectly conveyed data packets increases, and the total number of invalid transmitted packets increases. Therefore, this coexistence problem increases the retransmissions and latency of packets. Hence, it is essential to implement predictive mechanisms that allow avoiding and minimizing the impact of a channel coexistence problem for reliable packet delivery in BASNs. 

Nevertheless, to the best of our knowledge, few bio-inspired research efforts have been exploited to improve routing performances using distributed communication methods. For example, the research in [25] proposes a bio-inspired, multi-objective routing protocol to optimize the routing performance in BASNs. The proposed scheme achieves packet transmissions with no excessive collision, which successfully ensures both low energy consumption and high data transmission rates in the network. However, the memory required of the proposed scheme to store node topology information and to schedule neighbor information is large. Similarly, the work in study [26] also presented a bio-inspired routing protocol to find the optimal routing path for reliable data delivery to the sink. However, the proposed protocol does not consider a complete collision avoidance mechanism in BASNs. Table 1 shows the comparison of different routing schemes developed for BASNs.

## 3. Challenges and Research Motivations

In BASNs, the biosensors need to be extremely tiny in order to be wearable and implantable. Large on-body or in-body biosensors will heavily impact the daily lives, or even body functions, of patients. This size requirement imposes strict limitations on the battery size of the nodes. However, these nodes are expected to continuously work for several days. So, the biosensor nodes need to be extremely energy efficient. The main functions in which the energy of a sensor node is consumed are data collection from the body, data processing, and data transmission. In typical biomedical applications, energy consumption is dominated by the biosensors’ radio units during data transmission, which is highly dependent on communication links between nodes [27]. So, data need to be transmitted with extreme reliability, in terms of latency, packet error rates, and packet delivery rates, and the energy efficiency requirements need to be managed wisely since the signal collected and transmitted can be life-critical. The data collected in dynamic and frequently changing environments are mostly time-dependent and have temporal validity, within which the information should be transmitted to remote command centers with acceptable loss rates and in time to ensure effective and timely decision making. Thus, data freshness is one of the key issues that should be provided since collections are made in discrete points in time. Therefore, timely delivery is a crucial requirement since the data generated by the body sensors must be delivered in real-time to the destination in order to be processed and analyzed before the deadline; otherwise, the packet will no longer be useful [28]. To this end, the design of a BASN system requires coping with fast-changing environments in a responsive and real-time manner in healthcare applications, which generates heterogeneous traffic in the network. In addition, because of the relatively small antennas and simple energy-efficient designs, the receivers’ sensitivity level is not high either. If severe attenuation occurs around or within the human body, it may cause transmission outages due to poor link quality. Moreover, the received signal strength is mostly affected by unpredictable user body movements, which effects the wireless signals and leads to excessive rerouting due to poor links between nodes in the network.

Furthermore, because of the broadcast nature of the wireless medium and the limited radio spectrum, co-located BASNs may temporarily be exposed to a significant amount of interference in the network. Also, interference occurs when multiple body sensors come close together at distinct locations. In these cases, the coordination strategy designed may fail or reduce the communication performance of the entire system, which leads to low packet delivery rates in the network. To tackle these issues, several routing protocols have been proposed for BASNs in the literature (see Section 2 for details). The main purpose of the existing research work is to achieve reliable and energy-efficient wireless communications among the body sensors and to prolong the network’s lifetime. Some of these designs serve as a foundation for the BASNs, such as the idea of an energy-efficient packet forwarding mechanism [29]. However, most BASN studies face common issues, such as they do not consider the link quality, which results in excessive packet losses in the network. In addition, BASN nodes always prefer to choose the shortest path during packet forwarding, which may balance energy consumption at the individual node but degrades the overall energy consumption performance of the network. Moreover, intermittent network connectivity over the shortest paths may delay the transmission of critical and urgent data, leading to the loss of sensor readings and an increased number of fatalities faced by groups of users in harsh environments, and it may, in consequence, hamper communication efficiency. Furthermore, if the load balance is not considered, the nodes located in the center of the body and closer to the sink will face congestion issues. Besides, these schemes, because of a lack of multiobjective features, can only achieve one objective at the expense of others in the given time. Thus, these schemes cannot maximize the performance and solve the challenges associated with monitoring the human body; thus, they are quite vulnerable to body channel fluctuations in BASNs. Hence, a novel routing protocol is greatly needed for BASN-based health monitoring applications. 

All aforesaid facts motivated our research to develop a novel, multiobjective, lion mating optimization inspired, clustering-based routing protocol that simultaneously supports various Quality of Service (QoS) requirements for BASN-based IoT health monitoring applications. In the proposed scheme, the entire routing problem has been modeled using mixed-integer linear programming (MILP). This research has the following main contributions:

- A novel, multiobjective lion mating optimization algorithm is proposed to avoid local search problems during tackling the various objectives in the given problem search space.
- A multiobjective, lion mating optimization based routing mechanism is proposed to provide robust, reliable, and energy-efficient delivery of patient data to the medical data center in dynamic situations, where doctors or autodiagnostic systems can react to abnormal situations.
- Extensive simulation studies are performed using MATLAB 9.5 (R2018b) to validate the performance of the proposed scheme against the existing routing protocols designed for BASN-based health monitoring applications.

## 4. Proposed SARP Routing Protocol for BASNs

The protocol design details are given in the following sections.

### 4.1. Network Model and Assumptions

**Figure 1:**
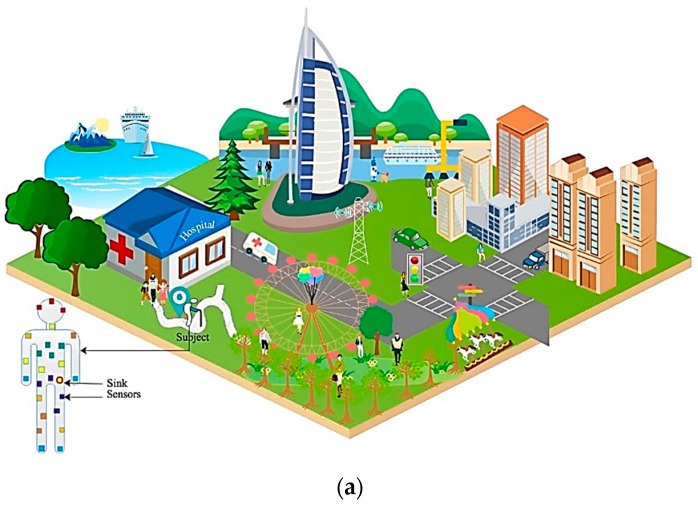

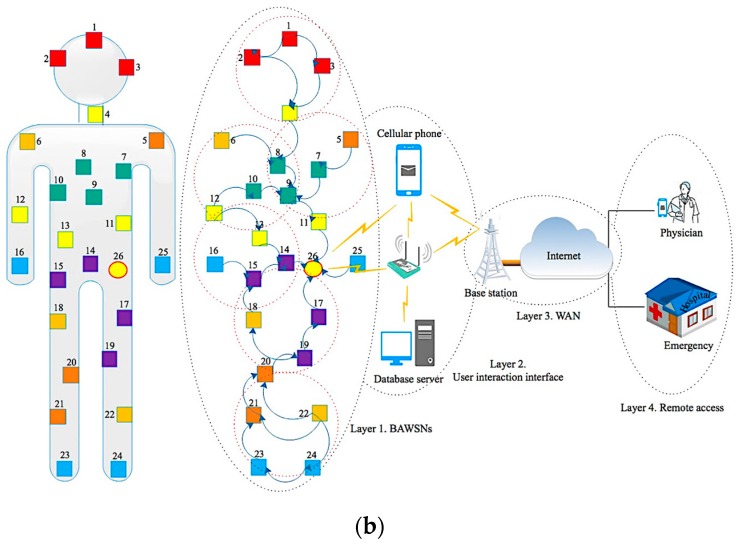
(**a**) A human with implanted biosensors in a remote location. (**b**): Network model for the SARP routing scheme. In which, BAWSNs and WAN indicates the Body Area Wireless Sensor Networks and Wide Area Network, respectively.

An illustration of the proposed wireless body area sensor network model for healthcare monitoring systems is given in Figure 1a,b. It can be seen from Figure 1b that a mobile body is in the standing position with arms hanging along the side, and there are various types of biosensors, such as electromyography sensors (nodes 6, 18, and 22), electroencephalogram sensors (nodes 1, 2, and 3), Arduino pulse sensors (7, 8, 9, and 10), temperature sensors (nodes 4, 11, 12, and 13 ), pressure sensors (16, 23, 24, and 25), osteoporosis sensors (nodes 14, 15, 17, and 19), motion sensors (nodes 5, 20, and 21), and one coordinator (node 26), on the surface of the human body. Different colors show the various types of biosensors located on the patient’s body. The coordinator is located at the left hip pocket, and the sensors are located at the head, shoulder, chest pocket, abdomen, left wrist, right wrist, right ankle, knees, calf, and so on. These biosensors continuously capture large quantities of life signals from the human body and transmit the information directly, or via relay nodes, to the sink. The role of the coordinator, which acts as a sink node for the biosensors, also acts as a gateway for communicating with the outside world. Thus, it is responsible for controlling the network, collecting all sensor data from the elementary sensors, and then relaying it to its destination in a prompt and reliable way. The coordinator role is of a personal digital assistant that communicates with the medical care center through wireless networks, such as a cellular network or a local area network connected to the wide-area network.

In the monitoring process, the end nodes located far away employ intermediate nodes to act as relay nodes to convey information to the sink. The relay nodes fulfill the functions of collecting and monitoring data as well. Once the coordinator collects the data from the sensors, the collected information is forwarded through the internet-enabled sink to the medical data center, where doctors or auto-diagnostic systems react to abnormal situations. The nodes in the wireless body area sensor network have short-range communication capabilities and are equipped with limited power, processing, and storage units. In sum, the body area sensor network is composed of three types of nodes, namely, biosensors, sink, and the relays. In simulation studies, we assumed the following: The deployed biosensor nodes on the human body are associated with a unique identifier by which they can be distinguished from others. Since the sensors are deployed deterministically, they have a fixed location and distance with the neighboring nodes along the surface of the human body in spite of body gestures. Thus, each node knows its location and are neighbors only if they are in the communication range of each other. The deployed sensors are equipped with equal initial power, memory, computation, and communication capabilities. The packet collision avoidance mechanism, called time division multiple access (TDMA), is used for collision-free channel access in BASNs. Finally, a remote user can access, monitor, and reconfigure the biosensors located on the human body via one of the communication technologies such as 4G, Ethernet, or WiFi.

### 4.2. Bio-Inspired Computing and Optimization Problems

Optimization is a procedure to find the most appropriate solution to the given problem of interest. There exist two main categories of optimization problems, namely, the combinatorial optimization problem and continuous optimization problem. The combinatorial optimization problem usually employs discrete variables, while the continuous optimization problem includes continuous variables of a problem. On the other hand, deterministic and stochastic algorithms are two main optimization methods. The deterministic algorithms require a small number of iterations to provide good efficiencies for certain problems. However, they face the problem of being trapped in local optima. On the contrary, stochastic algorithms can escape from the local optima by employing randomness in their strategies to search more regions on a global scale. Therefore, this technique offers a set of alternative solutions from the same initial points for each individual involved in the optimization process. The local search improves a candidate solution until advances are identified, while randomization avoids the solution being trapped into local optima. Exploration and exploitation are two major components in each metaheuristic algorithm search process. Some of the most popular metaheuristic algorithms include the genetic algorithm (GA) [30], animal migration optimization (AMO) [31], grey wolf optimization (GWO) [32], lion optimization algorithm (LOA) [33], and many others. In GAs, natural selection is simulated by a stochastic selection procedure. Each solution is allowed to regenerate based on its “fitness”. The variation process imitates the natural capability to create new generations by employing crossover and mutation. Here, crossover operates on various portions of chromosomes through the process of switching over. Mutations are accomplished by flipping a single, randomly selected gene within a chromosome. The main advantages of these algorithms include the low probability of entrapment into local modes and faster convergence due to appropriate information-sharing during optimization. 

#### 4.2.1. Multiobjective Problems (MOPs)

Many different applications in engineering, science, and industry have a considerable degree of complexity, and sometimes this complexity might be based on the existence of various conflicting objective functions, which must be simultaneously optimized. These kinds of problems are the so-called multiobjective optimization problems (MOPs). However, in everyday life, most optimization problems are not static in nature and usually have at least one objective that can change over time. Most researches focus on either static, multiobjective optimization or dynamic, single-objective optimization. However, the literature reveals that not much research has been done on dynamic multiobjective optimization (DMO) [34]. There are only a few studies on multiobjective optimization algorithms in different areas of study, such as in transportation, manufacturing, scheduling, systems engineering, and so on, because of their ability to successfully capture different and possibly conflicting goals of decision-makers [35]. However, some of the above-mentioned mechanisms when designed for multiobjective optimization problems have several limitations, and this introduces diversity during the optimization process, which usually depends on the ability of the optimization algorithm. Sometimes, such algorithms present difficulties in tracking the new positions on the Pareto optimal front. Furthermore, they may not efficiently work when the changes in the problem are severe or fast. The use of multiple populations in the design of existing multiobjective schemes can affect the performance of the optimization algorithm. Approaches based on prediction mechanisms depend on how well the predictors are trained. The use of memory-less approaches has the disadvantage of generating abundant information and may not necessarily promote diversity. Besides, these optimization algorithms are mostly designed to achieve a single objective as a trade-off with others and, therefore, cannot be directly implemented to achieve a set of objectives in the given problem search space instantaneously. Moreover, there are several mathematical programming methods that have shown to be effective for solving MOPs. However, there are cases where these methods cannot guarantee that the solution obtained is optimal. Also, some mathematical programming methods can be inefficient or even inapplicable for particular problems. Furthermore, one of the most important features that should be considered for the design of dynamic optimization algorithms is that such algorithms must present not only a fast convergence level but also a good mechanism to promote diversity. 

#### 4.2.2. Multiobjective Lion Mating Optimization Algorithm (MLOA)

The traditional LOA is inspired by social behaviors of lions in a habitat. Lions are social cats belonging to the family Felidae living in open savanna. Lions are primarily nocturnal or crepuscular with two classes of social organization, namely, residents and nomads. The resident type includes several generations of females and their cubs and a few males who live in a group called pride. In the nomad types, lions and lionesses who leave out their maternal pride live either in pairs or singularly. Thus, a lion or lioness may change their lifestyle between residents and nomads. In the habitat, each pride has a well-defined territory where the lions proclaim their territory by roaring and strictly defend against intruding lions entering their territory. In a pride, the lionesses are the main hunters who work in a group together to hunt for food [36]. They spread out and surround potential prey from different directions and take down prey with an instant attack. The lions also hunt prey together with lionesses in some areas; however, the main job is to protect the pride. Successful coordination among lions and lionesses in a hunting group can lead to a high probability of triumph in lion hunts. The lions and lionesses become tired after running short distances and pay no attention to the wind direction during hunting the prey. However, the wind may carry their scent to their prey, which lets the prey run away from the hunters, and also slows down their running speed during hunting. The lions spend most of their time resting in their pride for various reasons such as a lack of prey, to avoid the heat, and to conserve energy for the hunting day. 

During periods of rest, the lions and lionesses have several opportunities for social behavior such as chafing their heads together, playing, and sleeping in groups, which are important for fortifying their social relationships. During the mating period, lionesses may mate with multiple lions several times. In the evolution process, nomad lions may try to take over a pride and kill all the new cubs to avoid competition and to mate with the lionesses to produce their cubs [37]. The survived male cubs, when they reach maturity, become a nomad and have less influence than the resident males in a pride. However, if a strong nomad young male succeeds to drive out the resident male, then it becomes the resident lion of the pride. These nomad cats move randomly to find a better place in the habitat and also to hunt the prey, similarly to resident cats. Lionesses may switch their lifestyles between residents and nomads and migrate from one pride to another pride. Consequently, the weakest lion will die or be killed in a case of competition or lack of food in the pride. This entire process repeats their whole life until they die. However, the LOA cannot be directly applied for the MOPs in BASNs. To make it suitable for MOPs in BASNs, the basic LOA requires critical modifications at different protocol stack levels to make it suitable for MOPs in BASNs. Therefore, some necessary modifications—such as a Pareto search; a dynamic mating model; prey hunting location information, speed, and direction of hunting; search boundary limitations; and survival of the best to avoid local search problems—have been made in the basic LOA to adapt it to the BASN-based MOPs in in the proposed scheme.

Because of the aforesaid reasons, the use of metaheuristics in LMOA is a promising solution to solve MOPs in WBASNs. The LMOA is a kind of stochastic, nature-inspired, metaheuristic method that uses a kind of randomization to search a set of solutions. The proposed multiobjective LMOA provides the best solution among the available that contains a set of objectives to achieve a target in BASNs. In the proposed scheme, the fitness function and previous history prevents the algorithm from being trapped in a local search space during optimizing the given multiple problems. In LMOA, maintaining population diversity is a very important task. If the population diversity is lost prematurely, then tracking new, optimal positions in the environment becomes more difficult. In the LMOA, various GA selection operators, such as crossover and mutation operators, enhance the algorithm’s population diversity and the good performance of the scheme, as algorithm optimization promotes good convergence in the BASNs. Also, the survival selection mechanism plays an important role to determine the quality of the solutions that can survive through the optimization process. Different survival selection mechanisms have been proposed as being the most popular Pareto-based selection mechanism. It can break the tie among different objectives by generating the precise Pareto front to solve various problems in BASNs. However, if the problem is combinatorial, generating precise Pareto fronts can be challenging. In this study, we focused on a multiobjective set, covering problem using a decomposition method [38] for generating the precise Pareto front. Particularly, the decomposition method first divides the problem into a set of subproblems, then it generates the exact Pareto fronts of these subproblems, and, finally, it uses the subproblem Pareto fronts to acquire the frontier of the original problem. 

The rationale behind this decomposition approach is mainly two-fold. First, the decomposition method requires solving single-objective combinatorial problems to determine a Pareto efficient solution. The decomposition method requires generating Pareto efficient solutions for the decomposed subproblems, which have significantly smaller feasible regions than the feasible region of the main problem. Therefore, solving the single-objective problems for generating a Pareto efficient solution of a decomposed subproblem is relatively easier than solving the single-objective problem for generating a Pareto efficient solution of the main problem. Therefore, generating all of the Pareto efficient solutions of a subproblem is relatively easier than generating all of the Pareto efficient solutions of the main problem. Consequently, making these single-objective problems easier can improve the computational time. Secondly, the single-objective combinatorial problems that need to be solved become more difficult to solve after generating each Pareto efficient solution, as most of the exact methods need to iteratively ensure that a different Pareto efficient solution is generated. This, in turn, adds more variables and/or constraints to the single-objective combinatorial problem to be solved at each iteration and, therefore, increases the computational time, especially when the Pareto front is large. The decomposed problems with the decomposition approach might tend to have smaller Pareto fronts; therefore, generating all of the Pareto efficient solutions of a subproblem is relatively easier than generating all of the Pareto efficient solutions of the main problem. In this study, we focused on generating alternative solutions, particularly, Pareto efficient solutions for the problem of interest. A solution is a Pareto efficient when there are no other solutions and is superior in terms of all of the objective functions. Consequently, the key objective function $\left(\phi_{SARP}\right)$ of the proposed scheme in BASNs is to optimize the performance of each lion in each round of the simulation, which is defined in Equation (1) as

(1) $$\phi_{SARP}=\int_{b_{\ell }}^{b_{𝓊}}\left(\underset{\forall ℒ\left(𝒾\right)\in N}{\min}\sum_{𝒾}^{𝓍}{\left(E_{𝒸}+D_{ℯ}\right)}^{𝒾}+\underset{\forall ℒ\left(𝒾\right)\in N}{\max}\sum_{𝒾}^{𝓍}{\left(P_{𝒹𝓇}+J_{𝓅}\right)}^{𝒾}\right).$$

The entire working mechanism of the proposed algorithm is divided into different phases. All the notations used in mathematical modeling are defined in Table 2.

• Initialization

In the proposed algorithm, each solution in the solution space is called ‘lion’. First, an initial population is initialized randomly over the 𝓃-dimensional search space where the entire lion population (N) is divided into resident lions $\left({ℛ}_{ℒ}\right)$ and nomad lions $\left(N_{ℒ}\right)$, such that $N={ℛ}_{ℒ}+$ $N_{ℒ}$. The resident population lives in the form of groups $G_{𝒾}\left({ℛ}_{ℒ}\right)$ and consists of male lions (ℳℒ), female lions (ℱℒ), and cubs $\left({ℒ}_{C}\right)$, including both male cubs $\left({ℒ}_{ℳC}\right)$ and female cubs $\left({ℒ}_{ℱC}\right)$. Similarly, the nomad population contains a set of males, females, and cubs, including both male cubs and female cubs. In each pride, the female lion population is chosen around $\%P_{ℛℱ}$, while the rest are males $\%P_{ℛℳ}$. However, this ratio is around $1-\left(P_{ℛℱ}+P_{ℛℳ}\right)$ in nomad lions and vice versa. This can be numerically indicated as

(2a) ∀𝒾={1,2,…,𝓍}; ∀𝒿={1,2,…,𝓎}; ∀𝓀={1,2,…,𝓏};

(2b) $$\mathrm{Lions}\;\mathrm{population}:\;N=\left\{{ℒ}_{1},{ℒ}_{2},\ldots ,{ℒ}_{𝓃}\right\}\;\mathrm{or}\;N={ℛ}_{ℒ}\left(N_{ℳℒ}+N_{ℱℒ}\right)+N_{ℒ}\left({ℛ}_{ℳℒ}+{ℛ}_{ℱℒ}\right);$$

(2c) $$\mathrm{Resident}\;\mathrm{lions}:\;{ℛ}_{ℒ}=\left\{{ℛ}_{ℒ1},{ℛ}_{ℒ2},\ldots ,{ℛ}_{ℒ\left(𝓀\right)}\right\},$$

(2d) $${ℛ}_{ℳℒ}=\left\{{ℛ}_{ℳℒ1},{ℛ}_{ℳℒ2},\ldots ,{ℛ}_{ℳℒ\left(𝓃\right)}\right\},$$

(2e) $${ℛ}_{ℱℒ}=\left\{{ℛ}_{ℱℒ1},{ℛ}_{ℱℒ2},\ldots ,{ℛ}_{ℱℒ\left(𝓃\right)}\right\};$$

(2f) $$\mathrm{Nomad}\;\mathrm{lions}:\;N_{ℒ}=\left\{N_{ℒ1},N_{ℒ2},\ldots ,N_{ℒ\left(𝓀\right)}\right\},$$

(2g) $$N_{ℳℒ}=\left\{N_{ℳℒ1},N_{ℳℒ2},\ldots ,N_{ℳℒ\left(𝓃\right)}\right\},$$

(2h) $$N_{ℱℒ}=\left\{N_{ℱℒ1},N_{ℱℒ2},\ldots ,N_{ℱℒ\left(𝓃\right)}\right\};$$

(2i) $$\mathrm{Lion}\;\mathrm{cubs}:\;{ℒ}_{C}=\left\{{ℒ}_{C1},{ℒ}_{C2},\ldots ,{ℒ}_{C\left(𝓀\right)}\right\},$$

(2j) $$C_{ℳℒ}=\left\{C_{ℳℒ1},C_{ℳℒ2},\ldots ,C_{ℳℒ\left(𝓃\right)}\right\},$$

(2k) $$C_{ℱℒ}=\left\{C_{ℱℒ1},C_{ℱℒ2},\ldots ,C_{ℱℒ\left(𝓃\right)}\right\};$$

(2l) $$\mathrm{Group}\;\mathrm{of}\;\mathrm{lions}:\;G_{𝒾}\left({ℛ}_{ℒ}\right)=\left\{{ℛ}_{ℒ}|N_{ℒ}=ℳℒ1,ℳℒ2\ldots ;ℱℒ1,ℱℒ2,\ldots ;:ℳℒ\left(𝓃\right)|ℱℒ\left(𝓃\right)\;\right\}.$$

• Prey Hunting

In the searching process, each lion visits randomly and marks its best-visited position. Then, based on the best-visited locations by its members, each pride’s territory is defined in the search space. In each pride, the female and male hunters have specific strategies to look for the prey. They encircle the prey and catch it in order to provide food for their pride. The stalking behavior of these hunters in a group is divided into three basic types, namely, right-wing, center, and left-wing. These three types of hunters in a ${𝓃}_{𝓋𝒶𝓇}$ dimensional space can be numerically indicated as

(3a) $$ℋ\left({𝓃}_{𝓋𝒶𝓇}\right)=\left[\begin{array}{c} {ℋ}_{𝒾}\\ {ℋ}_{1}\\ {ℋ}_{2}\\ \vdots \\ \underset{Left}{\underset{︸}{{ℋ}_{𝓍}}} \end{array}\;\begin{array}{c} {ℋ}_{𝒿}\\ {ℋ}_{1}\\ {ℋ}_{2}\\ \vdots \\ \underset{Middle}{\underset{︸}{{ℋ}_{𝓎}}} \end{array}\;\begin{array}{c} {ℋ}_{𝓀}\\ {ℋ}_{1}\\ {ℋ}_{2}\\ \vdots \\ \underset{Right}{\underset{︸}{{ℋ}_{𝓏}}} \end{array}\right],$$

where ${ℋ}_{𝒾}$, ${ℋ}_{𝒿}$, and ${ℋ}_{𝓀}\in {ℋ}_{𝒽}$, and ${ℛ}_{ℱℒ}$ and $N_{ℱℒ}$ $\in \;ℱ{ℒ}_{𝒾}$.

The hunters located in the center have the highest fitness values compared to the other two wing groups. The initial fitness value of each lion f(ℒ) in the ${𝓃}_{𝓋𝒶𝓇}$ dimensional optimization problem can be statistically shown as

(3b) $$f\left(ℒ\right)=\left\{f\left({ℒ}_{1}\right),f\left({ℒ}_{2}\right),\ldots ,f\left({ℒ}_{𝓃}\right){𝓃}_{𝓋𝒶𝓇}\right\}.\;$$

During encircling the prey, each hunter optimizes its location by considering its current and previous positions along with the positions of members of the group. Therefore, the hunters attack from opposite sites in the search space to hunt the prey. This opposition-based learning [39] makes them eligible to intelligently hunt the prey and, therefore, is an effective approach for solving optimization problems. The position of each hunter during optimization is saved in the following matrix.

(3c) $$f{\left({ℋ}_{𝒾}\right)}_{{𝓃}_{𝓋𝒶𝓇}}=\left[\begin{array}{c} {ℋ}_{1,1}\\ {ℋ}_{2,1}\\ \vdots \\ \vdots \\ {ℋ}_{𝓃,1} \end{array}\begin{array}{c} {ℋ}_{1,2}\\ {ℋ}_{2,2}\\ \vdots \\ \vdots \\ {ℋ}_{𝓃,2} \end{array}\begin{array}{c} {ℋ}_{1,𝒹}\\ {ℋ}_{2,𝒹}\\ \vdots \\ \vdots \\ {ℋ}_{𝓃,d} \end{array}\right].$$

Consequently, the hunters, one after another, are selected randomly to attack the prey located in the center of hunters (i.e., $P_{𝒾}=\sum {ℋ}_{𝒽}$). During hunting, if a prey escapes from the hunters, then the new position of the prey can be computed as

(3d) $$P_{𝒾\left(\ell \right)}\left(𝓃ℯ𝓌\right)=P_{𝒾\left(\ell \right)}+𝓇\left(0,1\right)\times \left(P_{𝒾\left(\ell \right)}-H_{𝒽\left(\ell \right)}\right)\times \delta .$$

In the hunting process, each hunter improves its own fitness value, and the new positions of the left- and right-wings of hunters can be statistically indicated as

(3e) $$H_{\ell \left(𝒾,𝓀\right)}\left(𝓃ℯ𝓌\right)=\{\begin{array}{c} 𝓇\left(\left(2\times P_{𝒾\left(\ell \right)}-H_{𝒾\left(\ell \right)}\right),P_{𝒾\left(\ell \right)}\right),\;\left(2\times P_{𝒾\left(\ell \right)}-H_{𝒾\left(\ell \right)}\right)<P_{𝒾\left(\ell \right)}\\ 𝓇\left(P_{𝒾\left(\ell \right)},\left(2\times P_{𝒾\left(\ell \right)}-H_{𝓀\left(\ell \right)}\right)\right),\;\left(2\times P_{𝒾\left(\ell \right)}-H_{𝓀\left(\ell \right)}\right)>P_{𝒾\left(\ell \right)} \end{array}.$$

The new positions of hunters located in the center are updated as

(3f) $$H_{𝒿\left(\ell \right)}\left(𝓃ℯ𝓌\right)=\{\begin{array}{c} 𝓇\left(P_{𝒾\left(\ell \right)},H_{𝒿\left(\ell \right)}\right),\;H_{𝒿\left(\ell \right)}<P_{𝒾\left(\ell \right)}\\ 𝓇\left(H_{𝒿\left(\ell \right)},P_{𝒾\left(\ell \right)}\right),\;H_{𝒿\left(\ell \right)}>P_{𝒾\left(\ell \right)} \end{array}.$$

Also, the new position for a female lion moving towards a safe place is updated as

(3g) $$ℱ{ℒ}_{𝒾\left(\ell \right)}\left(𝓃ℯ𝓌\right)=ℱ{ℒ}_{𝒾}+2{𝒹}_{1}\cdot {𝓋}_{1}\cdot 𝓇\left(0,1\right)+{𝒹}_{2}\cdot {𝓋}_{2}\cdot \sigma \left(-1,1\right)\cdot 𝓉𝒶𝓃\left(\theta \right).$$

To find the best solution for optimization problems by considering the simulated social leadership and encircling behaviors, the best two lions in the search space for hunting the prey are considered as ${\vec{D}}_{𝒾}={ℋ}_{\ell \left(𝒾,𝓀\right)}\left(𝓃ℯ𝓌\right)|{ℋ}_{𝒿\left(\ell \right)}\left(𝓃ℯ𝓌\right)|ℱ{ℒ}_{𝒾\left(\ell \right)}\left(𝓃ℯ𝓌\right)$.

(3h) $${\vec{D}}_{1}=\left|{\vec{C}}_{1}\cdot {\vec{Xℋ}}_{𝒾}-{\vec{Xℋ}}_{𝓃}\right|;{\vec{D}}_{2}=\left|{\vec{C}}_{2}\cdot {\vec{Xℋ}}_{𝒿}-{\vec{Xℋ}}_{𝓃}\right|,$$

(3i) $${\vec{Xℋ}}_{1}=\left|{\vec{Xℋ}}_{𝒾}-{\vec{ℛ}}_{1}\cdot \left({\vec{D}}_{1}\right)\right|;{\vec{Xℋ}}_{2}=\left|{\vec{Xℋ}}_{𝒿}-{\vec{ℛ}}_{2}\cdot \left({\vec{D}}_{1}\right)\right|,$$

(3j) $${\vec{Xℋ}}_{𝓃}\left(𝓉+1\right)=\frac{{\vec{Xℋ}}_{1}+{\vec{Xℋ}}_{2}}{2},$$

where $\vec{ℛ}$ and $\vec{C}$ are computed as

(3k) $$\vec{ℛ}=2\vec{\alpha }\cdot {𝓇}_{1}-\vec{\alpha }\;\mathrm{and}\;\vec{C}=2\cdot {\vec{𝓇}}_{2}.$$

$\vec{C}$ and $\vec{ℛ}$ favor exploration, and they provide random weights to the prey for stochastically emphasizing (C>1 or C<1) the effect of prey in defining the distance as well as guarantee the exploration to assist the search agent to diverge from the prey, respectively. This random hunting behavior throughout the optimization procedure favors exploration and avoids the local optima problem, not only during initial iterations but also final iterations, in the problem space. Consequently, the success (S) of a hunter ${ℋ}_{𝒽\left(\ell \right)}$ to improve its best position (ℬ) at iteration 𝒿 in time ${𝓉}_{𝓀}$ in a group $G_{𝒾}\left({ℒ}_{\left(N\right)}\right)$ is defined as

(3l) $$S\left({ℋ}_{𝒽\left(\ell \right)}\right)=\{\begin{array}{c} 1,\;{ℬ}_{{𝓉}_{𝓀}\left({ℋ}_{𝒽\left(\ell \right)}\right)}^{𝒿}<{ℬ}_{{𝓉}_{𝓀}\left({ℋ}_{𝒽\left(\ell \right)}\right)}^{𝒿-1}\\ 0,\;{ℬ}_{{𝓉}_{𝓀}\left({ℋ}_{𝒽\left(\ell \right)}\right)}^{𝒽}>{ℬ}_{{𝓉}_{𝓀}\left({ℋ}_{𝒽\left(\ell \right)}\right)}^{𝒿-1} \end{array},$$

(3m) $$S\left({ℋ}_{𝒿\left(\ell \right)}\right)=\sum_{𝒾=1}^{𝓃}\sum_{𝒿=1}^{N}S\left({ℋ}_{𝒽\left(\ell \right)\in \;G_{𝒾}\left(ℛ{ℒ}_{\left(𝒿\right)}\right)}\right),$$

in which ${𝒹}_{1},{𝒹}_{2}$ is the distance between the female hunter location and the certain point chosen by the tournament selection $\left(T_{𝒾}^{𝓈𝒾𝓏ℯ}\right)$ among the pride’s territory, which can be computed as

(3n) $$T_{𝒾}^{𝓈𝒾𝓏ℯ}=\max\left(2,𝒸ℯ𝒾\ell \left(\frac{S\left({ℋ}_{𝒿\left(\ell \right)}\right)}{2}\right)\right).$$

Finally, the hunters catching the prey in the problem space are simulated using the following equation:

(3o) $$H_{𝒽\left(𝒾\right)}^{𝒿}=P_{𝒾\left(\ell \right)}^{𝒿}\;\;\;\;if\;\;f\left(P_{𝒾}^{𝒿}<H_{𝒽\left(𝒾\right)}^{𝒿}\right),$$

where 𝒿 shows the current iteration, and ℓ indicates the position of the 𝒾th prey at the 𝒿th iteration. After each successful hunt, the fitness and position of each lion in the pride are updated, which helps the hunters in the next rounds of hunting in the given problem space. The positions and fitness values of the resident hunters in the pride are stored in $ℳ{\left({ℛ}_{ℒ}\right)}_{𝒾}$ and $ℳf\left({ℛ}_{ℒ\left(𝒾\right)}\right)$ as

(3p) $$ℳ\left({ℛ}_{ℒ\left(𝒾\right)}\right)=\left[\begin{array}{c} {ℛ}_{ℒ}{}_{1,1}\\ {ℛ}_{ℒ}{}_{2,1}\\ \vdots \\ \vdots \\ {ℛ}_{ℒ}{}_{𝓃,1} \end{array}\begin{array}{c} {ℛ}_{ℒ}{}_{1,2}\\ {ℛ}_{ℒ}{}_{2,2}\\ \vdots \\ \vdots \\ {ℛ}_{ℒ}{}_{𝓃,2} \end{array}\begin{array}{c} {ℛ}_{ℒ}{}_{1,𝒹}\\ {ℛ}_{ℒ}{}_{2,𝒹}\\ \vdots \\ \vdots \\ {ℛ}_{ℒ}{}_{𝓃,𝒹} \end{array}\right],$$

and

(3q) $$ℳf\left({ℛ}_{ℒ\left(𝒾\right)}\right)=\left[\begin{array}{c} f\left(\left[{ℛ}_{ℒ}{}_{1,1},{ℛ}_{ℒ}{}_{1,2},\ldots ,{ℛ}_{ℒ}{}_{1,𝒹}\right]\right)\\ f\left(\left[{ℛ}_{ℒ}{}_{2,1},{ℛ}_{ℒ}{}_{2,2},\ldots ,{ℛ}_{ℒ}{}_{2,𝒹}\right]\right)\\ \vdots \\ \vdots \\ f\left(\left[{ℛ}_{ℒ}{}_{𝓃,1},{ℛ}_{ℒ}{}_{𝓃,2},\ldots ,{ℛ}_{ℒ}{}_{𝓃,𝒹}\right]\right) \end{array}\right]\;,$$

where ℳ is the matrix for saving the fitness of each hunter $f\left({ℋ}_{𝒾}\right)$, and f is the objective function. Similar to resident lions, the nomad lions and lionesses also move randomly in the problem search solution to avoid being trapped in local optima. They move towards a certain area of territory by ζ units as

(3r) ζ=𝓊(0,2×𝒹(ℳℒ𝒾,ℳℒ𝒿)·θ) where θ∈−π6,π6.

The new position of each nomad lion in the search is updated as

(3s) $$N_{𝒿\left(\ell \right)}\left(𝓃ℯ𝓌\right)=\{\begin{array}{c} N_{𝒾𝒿\left(\ell \right)},\;𝓇>\rho_{b\left(𝒾\right)}\\ {𝓋}_{𝓇},\;otherwise \end{array},$$

in which ${𝓋}_{𝓇},\;N_{𝒾\left(\ell \right)}$, and $\rho_{b\left(𝒾\right)}$ are, as randomly generated vector in the search space, the present location of 𝒾th nomad lion in the 𝒿th dimension, and the probability for each nomad lion independently can be calculated as

(3t) $$\rho_{b\left(𝒾\right)}=0.1+\min\left(0.5,\;\frac{\left(N_{𝒾\left(\ell \right)}-{ℬ}_{{𝓉}_{𝓀}\left(N_{𝒿\left(\ell \right)}\right)}^{𝒿}\right)}{{ℬ}_{{𝓉}_{𝓀}\left(N_{𝒿\left(\ell \right)}\right)}^{𝒿}}\right).$$

The positions and fitness values of the nomad hunters are stored in $ℳ{\left(N_{ℒ}\right)}_{𝒾}$ and $ℳf\left(N_{ℒ\left(𝒾\right)}\right)$ as

(3u) $$ℳ\left(N_{ℒ\left(𝒾\right)}\right)=\left[\begin{array}{c} N_{ℒ}{}_{1,1}\\ N_{ℒ}{}_{2,1}\\ \vdots \\ \vdots \\ N_{ℒ}{}_{𝓃,1} \end{array}\;\begin{array}{c} N_{ℒ}{}_{1,2}\\ N_{ℒ}{}_{2,2}\\ \vdots \\ \vdots \\ N_{ℒ}{}_{𝓃,2} \end{array}\;\begin{array}{c} N_{ℒ}{}_{1,𝒹}\\ N_{ℒ}{}_{2,𝒹}\\ \vdots \\ \vdots \\ N_{ℒ}{}_{𝓃,𝒹} \end{array}\right]$$

and

(3v) $$ℳf\left(N_{ℒ\left(𝒾\right)}\right)=\left[\begin{array}{c} f\left(\left[N_{ℒ}{}_{1,1},N_{ℒ}{}_{1,2},\ldots ,N_{ℒ}{}_{1,𝒹}\right]\right)\\ f\left(\left[N_{ℒ}{}_{2,1},N_{ℒ}{}_{2,2},\ldots ,N_{ℒ}{}_{2,𝒹}\right]\right)\\ \vdots \\ \vdots \\ f\left(\left[N_{ℒ}{}_{𝓃,1},N_{ℒ}{}_{𝓃,2},\ldots ,N_{ℒ}{}_{𝓃,𝒹}\right]\right) \end{array}\right].$$

• Cub Generation and Growth Procedure

The mating process begins after selecting the lioness and lion(s) to produce new cubs. This fertility evaluation process provides the productivity of the new female lion cubs and the male lion cubs and also produces an updated lioness, named as $ℱ{ℒ}_{𝒿}^{+}$, which may be obtained as

(4a) $$ℱ{ℒ}_{𝒿}^{+}=\{\begin{array}{ll} ℱ{ℒ}_{𝒿}^{+}, & \;if𝒿=\ell \;\\ ℱ{ℒ}_{1}^{+}, & otherwise \end{array},$$

where 𝓀 is a random integer, and $ℱ{ℒ}_{\ell }^{+}$ and $ℱ{ℒ}_{𝒿}^{+}$ are the ℓth and 𝒿th elements of $ℱ{ℒ}_{𝒿}^{+}$:

(4b) $$ℱ{ℒ}_{𝒿}^{+}=\min⎣ℱ{ℒ}_{𝒿}^{𝓂𝒶𝓍},\;𝓂𝒶𝓍\left(ℱ{ℒ}_{𝒿}^{𝓂𝒾𝓃},\nabla 𝒿\right)⎦,$$

(4c) $$\nabla 𝒿=⎣ℱ{ℒ}_{𝒿}+\left({𝓇}_{2}-0.5\right)\left(ℳ{ℒ}_{𝒿}^{𝒾}-{𝓇}_{1}ℱ{ℒ}_{𝒿}^{𝒾}\right)⎦,$$

where ∇ denotes the female update, and ${𝓇}_{1}$ and ${𝓇}_{2}$ denote the random integers between [0, 1]. On the other hand, the female and male offspring, $OS_{\left(𝒿\right)}^{1}$, can be indicated as

(4d) $$OS_{\left(𝒿\right)}^{1}=\beta \times ℱ{ℒ}_{𝒿}+\sum \left(\frac{\left(1-\beta \right)}{\sum_{𝒾=1}^{𝓃}G_{𝒾}\left({ℛ}_{ℳℒ\left(𝒾\right)}\right)}\right)\times ℳ{ℒ}_{𝒿}^{𝒾}\times S_{𝒾},$$

(4e) $$OS_{\left(𝒿\right)}^{2}=\left(1-\beta \right)\times ℱ{ℒ}_{𝒿}+\sum \left(\frac{\beta }{\sum_{𝒾=1}^{𝓃}G_{𝒾}\left({ℛ}_{ℳℒ\left(𝒾\right)}\right)}\right)\times ℳ{ℒ}_{𝒿}^{𝒾}\times S_{𝒾}),$$

in which $S_{𝒾}$ is 1 only if a male lion is chosen for mating, otherwise it equals 0. Consequently, this process uses two fundamental steps called crossover and mutation. We used a random arithmetic crossover $\left(C_{𝓇}\right)$ with crossover probability 0.98 and 0.99 between two parents $\left(ℱ{ℒ}_{𝒿},ℳ{ℒ}_{𝒿}\right)$ to produce four cubs ${ℒ}_{C\left(𝓀\right)}$ to increase the diversity in the population as

(4f) $$\rho_{b}\left({ℒ}_{C}\right)=\;\gamma_{\rho_{b}}^{\circ }ℳ{ℒ}_{𝒿}^{𝒾}+{\bar{\gamma }}_{\rho_{b}}^{\circ }ℱ{ℒ}_{𝒿}^{𝒾},$$

where $\gamma^{{}^{\circ}}$ denotes the crossover mask, and $\bar{\gamma }$ indicates one’s complement of γ. The mutation method $\left({𝓂}_{𝓇}\right)$ swaps the genes at different points between chromosomes, randomly. The mutation operator with a probability between 0.01 and 0.05 is applied to ${ℒ}_{C\left(𝓀\right)}$ at multiple points to maintain the diversity and help the algorithm out of local optimum solutions. This can be numerically indicated as

(4g) $${𝓂}_{𝓇}{}^{1}=OS_{\left(𝒿\right)}^{1}+\;\lambda *\left(\overset{\ldots }{OS_{1}^{1}},\hat{OS_{2}^{2}},\ldots ,OS_{4}^{1},\ldots ,OS_{𝓀}^{1}\right),$$

(4h) $${𝓂}_{𝓇}{}^{2}=OS_{\left(𝒿\right)}^{2}+\;\lambda *\left(OS_{1}^{2},\hat{OS_{2}^{1}},\ldots \bar{,OS_{4}^{2}},\ldots ,OS_{𝓀}^{2}\right),$$

where λ∈[0, 1]. This process produces the equivalent number of new cubs. Then, the fitness of each individual is computed based on the fitness functions as

(4i) $$fit_{𝒿}^{𝓉}=\frac{fit_{𝒾}^{𝓉}}{\sum_{𝒿=1}^{𝓃}fit_{𝒿}^{𝓉}}.$$

Then, the best individual of the next generation (ℊ+1) is selected from the current generation (ℊ). In the cub growth process, a cub replaces the old cub if the mutated cub is strong, and then the new cub’s age is enhanced by one. Thus, the newly survived cubs essentially can affect the convergence rate to minimize the problem and can be summarized as

(4j) $$Cubs{\left({ℒ}_{𝒾}\right)}^{ℊ+1}=\{\begin{array}{cc} {ℒ}_{𝒾}^{ℊ+1} & if\;{\left({ℒ}_{𝒾}\right)}^{ℊ}<fit{\left({ℒ}_{𝒾}\right)}^{ℊ+1}\;\\ {ℒ}_{𝒾}^{ℊ} & otherwise \end{array}.$$

In the next step, the male cubs $C_{ℳℒ}$ and the female cubs $C_{ℱℒ}$ are extracted based on their first and second-best fitness values, as ${ℒ}_{C}\left(𝓃ℯ𝓌\right)$, which numerically is indicated as

(4k) $${ℒ}_{C}\left(𝓃ℯ𝓌\right)=C_{ℳℒ-{ℒ}_{C}}+\alpha \exp\left[-\gamma {𝒹}_{C_{ℳℒ}-C_{ℱℒ}-{ℒ}_{C}}^{2}\right]\left(C_{ℱℒ-{ℒ}_{C}}-C_{ℳℒ-{ℒ}_{C}}\right)+\delta_{𝒸}\epsilon_{𝒸},$$

in which $\epsilon_{𝒸}$ and $\delta_{𝒸}$ show the vector drawn from a Gaussian distribution and parameter that controls size, respectively, for the selected $C_{ℳℒ}$ and $C_{ℱℒ}$ cubs, where their ages are set to 0. After this, the history of mating with different males is sorted from best $\left({𝒷ℯ𝓈𝓉}^{𝓉}\right)$ to worst $\left({𝓌ℴ𝓇𝓈𝓉}^{𝓉}\right)$ in decreasing order based on the fitness value in each iteration 𝓉. This can be numerically indicated as

(4l) $${𝓌ℴ𝓇𝓈𝓉}^{𝓉}=\underset{𝒿\in \left[𝒿=1,2,\ldots \;,\;N_{𝓈𝓅ℯ𝓇𝓂}\right]}{\min}fit_{𝒿}^{𝓉},$$

(4m) $${𝒷ℯ𝓈𝓉}^{𝓉}=\underset{𝒿\in \left[𝒿=1,2,\ldots \;,\;N_{𝓈𝓅ℯ𝓇𝓂}\right]}{\max}fit_{𝒿}^{𝓉}.$$

Thus, the history of each female lion mating with the males is kept for a particular time, which will be used in the next round of the mating process.

• Territorial Defense

Territorial defense constitutes the survival fight between nomads and residents, updates on the pride, and coalition between the nomad lions and lionesses. Territorial takeover usually happens in the pride when cubs grow to maturity and become stronger (i.e., ${ℒ}_{C}>A_{𝓂𝒶𝓍}$). Otherwise, the cubs’ growth process is repeated. This process assigns territory to the stronger ones, $C_{ℳℒ-{ℒ}_{C}}$ and $C_{ℱℒ-{ℒ}_{C}}$, to grow and develop rather than the weaker $ℳ{ℒ}_{𝒾}$ and $ℱ{ℒ}_{𝒿}$ ones. On the other hand, territorial defense also occurs between residents and nomads. The pride is updated by replacing ${ℛ}_{ℳℒ\left(𝒿\right)}$ with $N_{ℒ\left(𝒾\right)}$ only when ${ℛ}_{ℳℒ\left(𝒿\right)}$ is defeated in the territorial defense process by the nomads. In this scenario, the pride is updated by selecting only one nomad $N_{ℒ\left(𝒾\right)}$ such that $N_{ℒ\left(1\right)}$, if $N_{ℒ\left(𝒾\right)}\geq ℯ\cdot \varsigma_{\left(E\right)}$ is met, else $N_{ℒ\left(2\right)}$ will be selected in the pride. Thus, a winning nomad $N_{ℒ\left(𝒾\right)}$ is chosen as

(5a) $$f\left(N_{ℯ-ℒ\left(𝒾\right)}\right)<f\left({ℛ}_{ℳℒ\left(𝒿\right)}\right)$$

(5b) $$f\left(N_{ℯ-ℒ\left(𝒾\right)}\right)<f\left({ℛ}_{ℳℒ\left(𝒿\right)-{ℒ}_{C}}\right)$$

(5c) $$f\left(N_{𝒾-ℒ\left(𝒿\right)}\right)<f\left({ℛ}_{ℱℒ\left(𝒿\right)-{ℒ}_{C}}\right),$$

where $N_{ℒ\left(1\right)}$ is

(5d) $$N_{ℒ\left(1\right)}=\exp\frac{{𝒹}_{3}}{\max\left({𝒹}_{3},{𝒹}_{4}\right)}\cdot \max\frac{\left(f\left(N_{ℒ\left(1\right)}\right),f\left(N_{ℒ\left(2\right)}\right)\right)}{f\left(N_{ℒ\left(1\right)}\right)},$$

in which ℯ,$\;\varsigma_{\left(E\right)}$, ${𝒹}_{3}$, and ${𝒹}_{4}$ are, respectively, the exponential of unity, the influence factor that lies in [1, 0], the Euclidean distance between the pair $\left(N_{ℒ\left(1\right)},{ℛ}_{ℳℒ\left(𝒿\right)}\right)$, and the Euclidean distance between the pair $\left(N_{ℒ\left(2\right)},{ℛ}_{ℳℒ\left(𝒿\right)}\right)$. Consequently, the nomad lions with the least fitness value will be removed at the end of each iteration in order to keep the ratio of the lions in the population constant.

• Termination Criteria

Termination is the last phase of the algorithm and occurs when the best solution is found in the problem space. It also includes the defined maximum time, the number of iterations reached, and there are no more improvements in the solution after a number of iterations. 

### 4.3. Mapping LMO for BASNs

In this section, we mapped the lion mating optimization implementation for the sensor nodes in BASNs. The lions are the biosensor nodes. The territory is a region where biosensor nodes are located in the network. There are multiple territories in the network. In each territory, pride is a group of resident lions and lionesses that live together, and it means that the biosensors are located in the group in a region. The male resident lion is the cluster leader in a group, where the female resident lions are the member biosensors linked to the group leader in a group. The nomad male and female lions are the biosensor nodes, which are isolated but linked to at least one neighboring sensor node in the network. Mostly, these nodes are located near the boundary of the network. The prey means a biosensor node that has the required information for the neighboring nodes in the network. The hunters mean the biosensor nodes try to find the neighboring nodes that have the required information or are used for packet transmission. When female lions go hunting, it means that those biosensor nodes that are located in the middle of the region have a high probability to take part in the packet forwarding process, while the rest of the sensor nodes may sense and share their information with the neighboring nodes. The female and male lions attack from the left, right, and middle, meaning that the biosensor nodes located on the left, right, and middle try to communicate with the desired biosensor node in the region. The middle lion has a high probability to hunt, meaning that the sensor node that has a lower distance and angle to the neighboring node is selected for information transmission to avoid loops in the network. The distance between the lions means the distance between the biosensor nodes in the network. The resident lion or nomad lion that has unity and influence becomes the new leader of the group, meaning that a sensor node with a high residual energy and low transmission distance to its member nodes has a high chance of being selected as a cluster head in the next round in the pride. In the pride, a new male lion cub that matures may become the leader, which means that a node with a high residual energy, shorter distance, and smaller angle to the neighboring nodes may replace the existing cluster leader in the current round. Otherwise, it will join the cluster leader as a member node in the network. Similarly, during the packet forwarding process, a cluster leader that has a high residual energy and lower distance to the next hop towards the sink will be selected as a forwarding relay node in the network. The newly elected cluster leader is responsible for maintaining the history of the entire member nodes in a routing table, which improves the probability of creating stable clustering architecture in the entire network. In addition, each nomad and pride lion and lioness are responsible for maintaining the hunting history, cub generation, and territorial defense, meaning that each biosensor node is responsible for maintaining the packet transmission history, active neighboring nodes, residual energy, and distance information. This helps to ensure the quality of small-size cluster formations and packet forwarding over highly reliable links towards the sink in the network. The termination criteria are satisfied when a routing path over a set of cluster heads is established between the source and the sink in the network. The entire above-mentioned discussion has been summarized in Algorithm 1.

**Algorithm 1:** pseudo code for LMO in SARP.**Input:** Generate a random population of Lions, i.e., sensor nodes $\left(S_{𝒾}\right)$ in a search space $\left({ℛ}_{𝒾}\right)$
**Output:** Cluster-based routing solution for BASNs. **Procedure:** Initialize all sensor nodes with the parameters given in Table 3
1. Begin the iterative methodology and set the round number = ${𝓉}^{+}$2. While (number of generations) do3. Initialize all members of prides and nomad lions on the problem search space.4. Select a percentage of the nomads $N_{ℒ}$ and the resident lions ${ℛ}_{ℒ}$ such that $N={ℛ}_{ℒ}+$ $N_{ℒ}$.5. Both lioness and lion are selected for hunting a prey, randomly.6. For each lion do7. Compute the fitness8. End for9. If the fitness of a lion is greater than other lion in a pride Then10.Update the lion in the pride in the problem search space11.For each lion do12.Try mating behavior13.A percentage of nomad females mate with the best males14.In each pride, a ratio of sex rate (S) is applied to ℳℒ(𝒾) and ℱℒ(𝒿) to produce new cubs15.If no improvement in fitness value then16.Try territorial defense behavior17.If no improvement in fitness value then18.Try territorial takeover behavior19.This ratio is inverted in nomad lions.20.End if21.End if22.End while23.For each pride 24.Both of male and female search randomly in the search space. 25.Nomad males attack prides randomly. 26.End for 27.For each bride 28.Some randomly selected female emigrates from the pride and becomes a nomad. 29.End for 30.Each gender of nomad lions is sorted according to their fitness value. 31.The best females are distributed to the prides to fill the empty places of the immigrated females.32.Compare and swap the weak lions with best fitness values lions.33.Memorize the best solution34.While (next generation until stopping criteria not met) do35.Send control to line 1 such that 𝓉<𝓉+1, Else36.Return the best solution37.End procedure

### 4.4. Working Principle of Sensors in SARP

For a clear understanding, the entire working mechanism is explained below.

#### 4.4.1. Updating Neighbors with Recent Information

A self-optimized, bio-inspired routing protocol for quality-aware data gathering is required for the next generation of wireless body area sensor networks. The proposed scheme, by employing the various types of biosensors, senses the human body and sends this information in a collaborative manner to the remote physician for monitoring a patient’s health condition in real time. In this respect, the key objective is to establish a set of energy-efficient and highly stable routing paths over a set of highly stable clustering architectures to provide quality-aware data transmission to the static coordinator. The entire routing architecture of the proposed scheme is divided into two main phases, namely, cluster formation and routing path selection, over a set of group leaders between the source and destination in BASNs. The patient or the remote doctor initiates the data collection process on demand by sending multiple data collection messages (𝒸ℴℓℓℯ𝒸𝓉_𝒹𝒶𝓉𝒶) to the coordinator located on the human body. The coordinator, after successfully receiving the first 𝒸ℴℓℓℯ𝒸𝓉_𝒹𝒶𝓉𝒶 message, looks in its routing table for any available path to the destination node. The coordinator immediately performs route discovery and broadcasts multiple route discovery messages (𝒹𝒾𝓈_𝓇ℴ𝓊𝓉ℯ) to the neighboring biosensor nodes in its communication range only if a path to the destination is not present or is invalid. The 𝒹𝒾𝓈_𝓇ℴ𝓊𝓉ℯ message contains information of the coordinator identity, distance, and location on the body. After successfully receiving the 𝒹𝒾𝓈_𝓇ℴ𝓊𝓉ℯ, each biosensor node creates a new routing table only if it does not exist before for storing the coordinator information in its routing table, and it replies to the coordinator node via a reply message (𝓇ℯ𝓅_𝓂𝓈ℊ) only if it is a single hop away to the sink. The reply message (𝓇ℯ𝓅_𝓂𝓈ℊ) contains the identity, residual energy, distance information, and its location on the body. The coordinator, after receiving the 𝓇ℯ𝓅_𝓂𝓈ℊ message successfully from the sender, stores its identity and other information, and marks it a single hop neighbor in its neighboring routing table. 

Then, each biosensor, by considering the time intervals, broadcasts the received 𝒹𝒾𝓈_𝓇ℴ𝓊𝓉ℯ messages in its communication range and waits for the neighbor reply messages. The biosensor node which received the 𝒹𝒾𝓈_𝓇ℴ𝓊𝓉ℯ messages from the neighboring sender updates its routing table with the recent information only if the entry for a particular node exists in the routing table. Otherwise, a new entry is created for each neighboring biosensor in the routing table. After updating the table, each receiver biosensor replies via 𝓇ℯ𝓅_𝓂𝓈ℊ message to the sender node and waits for the acknowledgment message (𝒶𝒸𝓀𝓃ℴ_𝓂𝓈ℊ). Upon receiving the first reply message, each receiver biosensor waits for a distinct time to collect all other 𝓇ℯ𝓅_𝓂𝓈ℊ messages from the neighboring nodes. This waiting period is kept constant so that the maximum number of reply message can be collected before it expires. Consequently, the receiver biosensor node updates its routing table and sends the neighboring node information to the sink, which updates its information in its routing table and marks the new neighboring node with increasing hop number since it is away from the sink. Later, it broadcasts an 𝒶𝒸𝓀𝓃ℴ_𝓂𝓈ℊ message to the neighboring nodes with their hop count information in the network. Both the 𝓇ℯ𝓅_𝓂𝓈ℊ and 𝒶𝒸𝓀𝓃ℴ_𝓂𝓈ℊ messages contain similar information of the sender and receiver, including the node identity, energy level, distance, and current location on the body. However, some extra bits are included in the 𝒶𝒸𝓀𝓃ℴ_𝓂𝓈ℊ message in order to ensure the successful transmission over a link between the biosensors in the body area sensor network. This process repeats until each biosensor node and the sink have the information of neighboring nodes in the wireless body area sensor networks. 

Throughout the process, each biosensor node sets the neighboring hop count level values in both upward and downward directions in its routing table. This not only helps to provide the distance and location information of the biosensor nodes from the sink but also controls the packet loops in the network. In addition, during the message transmission process, an acknowledgment message is forwarded by the receiver to the sender node in order to ensure successful packet exchange over a link in the network. Moreover, packet collision is avoided throughout the message exchange process by considering the TDMA mechanism. 

#### 4.4.2. Dynamic Cluster Formation

At this stage, each biosensor node has updated information on the neighboring nodes as well as the coordinator in BASNs. Consequently, a node which has a high residual energy and lower distance to its neighboring biosensors, as well as to the sink, declares itself a group leader and broadcasts this message (ℓℯ𝒶𝒹ℯ𝓇_𝓂𝓈ℊ) to its neighboring nodes located in the region. The key aim of this message is to request neighboring biosensors for joining the sender as member nodes. The ℓℯ𝒶𝒹ℯ𝓇_𝓂𝓈ℊ message contains short information, like sender node identity and its priority, which is set to 1 for the cluster head in BASNs. Upon receiving the ℓℯ𝒶𝒹ℯ𝓇_𝓂𝓈ℊ message, the neighboring biosensors that already have the sender information update their status to 1 as a leader node in their routing table. Then, the interested biosensor nodes reply to the potential cluster leader via a leader acceptance message (ℓℯ𝒶𝒹ℯ𝓇_𝒶𝒸𝒸ℯ𝓅_𝓂𝓈ℊ) with its identity and location on the body. Then, each head node, upon receiving the ℓℯ𝒶𝒹ℯ𝓇_𝒶𝒸𝒸ℯ𝓅_𝓂𝓈ℊ acceptance message from the neighboring node, updates its information and sets the priority value to 0 for the member node in the routing table. Later, each cluster leader broadcasts a message (𝒸ℓ𝓊𝓈𝓉ℯ𝓇_𝓂ℯ𝓂𝒷ℯ𝓇) that contains the information of the member node’s identity and a certain time slot for each member node to communicate in BASNs. Thus, the entire region is partitioned into a set of predefined, highly stable, dynamic clusters in BASNs. Throughout this process, it is possible that a node may join two cluster leaders; however, it must have different time slots for communication in order to avoid packet loss and interference in BASNs. 

#### 4.4.3. Packet Forwarding Over a Set of Optimal Biosensors

The packet forwarding procedure begins when a biosensor node senses the events and wishes to send the data packet to its cluster head in BASNs. The cluster leader, after receiving the information, first looks in its routing table if a route exists, then it forwards the received data to the next hop cluster head relay node towards the sink. Otherwise, it sends a packet forwarding request (𝓇ℯℓ𝒶𝓎_𝓅𝒶𝒸𝓀ℯ𝓉𝓈) message to its neighboring cluster heads, using both low- and high-transmission powers, and waits for the 𝓇ℯ𝓅_𝓂𝓈ℊ message for a predefined time interval. The 𝓇ℯ𝓅_𝓂𝓈ℊ message contains the sender cluster leader identity, residual energy, location, and level information in the network. Each receiver cluster head, after successfully receiving the 𝓇ℯℓ𝒶𝓎_𝓅𝒶𝒸𝓀ℯ𝓉𝓈 request message from the neighboring cluster leader, marks its identity and hop count in decreasing order, as it is away from the sink in its neighboring routing table only if the entry for a particular node exists in the routing table. Otherwise, a new entry is created for each neighboring head node in the routing table. Only the interested cluster heads reply to the sender request via sending a 𝓇ℯ𝓅_𝓂𝓈ℊ message with their identity, residual energy, and level and distance information towards the sink. Then, the receiver cluster leader, upon receiving the 𝓇ℯ𝓅_𝓂𝓈ℊ message, computes the probability of each forwarder, updates its routing table with the priority information, and selects the best forwarder towards the sink. A cluster head with high residual energy and shorter distance to the sender and sink has a high probability of being selected as a forwarder node in BASNs. The receiver selects the potential forwarder randomly if more than one forwarder has the same probability. After selecting a potential forwarder, it sends a ready packet receive message (𝓇ℯ𝒶𝒹𝓎_𝓇ℯ𝒸_𝓂𝓈ℊ) to inform about the arrival of the packet. On packet arrival, at each forwarder, the routing table entries indicate that the data packets already have visited the sender cluster head, thus it avoids sending the same packet to a neighboring cluster head for the second time, which ensures that the discovered routing paths are loop-free in BASNs. This process repeats at each hop, and packets keep moving in the direction of the sink. As soon as the packets are received from the neighboring cluster head, the sink sends an 𝒶𝒸𝓀𝓃ℴ_𝓂𝓈ℊ message to the source cluster head that includes the identity of all relay nodes along a routing path in BASNs. Thus, a routing path over a set of cluster heads is verified in both upward and downward directions between the source and destination in the network. In the entire routing process, each cluster leader is responsible for maintaining the activities of its member nodes, neighboring cluster head nodes, and the list of routing paths, and it updates it periodically. This periodic information update provides a loop-free network in the discovered routes.

#### 4.4.4. Route Repair Procedure

Route maintenance occurs only when a forwarder node fails to convey data packets to the sink. The designed scheme instantaneously identifies the route breakdown, which is very common in BASNs. Route failure is identified when a sender cluster head has not received an acknowledgment message from the receiver cluster head in a predefined time interval. In case of a route failure, the sender cluster head first looks in its routing table for the alternative forwarding nodes, if they exist, then it selects the second-highest priority forwarder as the alternative route and sends a 𝓇ℯ𝒶𝒹𝓎_𝓇ℯ𝒸_𝓂𝓈ℊ message to inform about the arrival of packets. Simultaneously, it deletes the records relating to the failure node and updates the priorities of the rest of the remaining forwarders in the routing table. Otherwise, the sender cluster leader will start the route discovery process discussed above to establish a routing path towards the sink. As soon as the packets are received, the sink identifies the route changes and sends an 𝒶𝒸𝓀𝓃ℴ_𝓂𝓈ℊ message to the source cluster head, including the identity of all relay nodes along a routing path in BASNs. Thus, a new routing path over a set of cluster heads is verified in both upward and downward directions between the source and destination in the network. However, in case of a member node failure, the cluster head just discards that node from its member list and updates the routing table. In the entire routing process, each cluster head maintains the priority of the associated forwarders in the routing table, which keeps changing as time passes until the forwarder battery is drained. This priority helps the forwarder to distribute packets relaying the responsibility among different relaying nodes in order to balance the network traffic and energy consumption load in BASNs. The designed scheme adopts a simple procedure to tackle route failure problems and, therefore, avoids high energy consumption issues due to low overheads in BASNs. 

In the proposed scheme, the entire body area network is represented as a graph G(V,ℰ), in which the vertices $V=V_{S_{𝒾}}\cup V_{S_{𝒾}\left(\ell \right)}\in S_{1}$ and the edges $ℰ=V_{S_{𝒾}}\subseteq V_{S_{𝒾}\left(ℒ\left(𝒿\right)\right)}\in ℒ\left(𝓃\right)$, such that $S_{𝒾}$ = {$S_{𝒿}$: ($S_{𝒾}$,$\;S_{𝒿}$) ∈ ℰ } represents the sensor nodes $S_{𝒾}$, and their wireless links ℒ(𝒿) located in a particular region $V_{S_{𝒾}\left(\ell \right)}$ belong to the sink $\left(S_{1}\right)$. The packets generated by each sensor node are divided into rounds ℛ where each round ${ℛ}_{𝒾}$ consists of time frames $T_{f}$, such that each frame ${𝓉}_{f}\subset T_{f}$. At the beginning of each round, the residual energy of the sensor node is ${ℛ}_{ℯ\left(𝒾\right)}$, which decays to $\xi {ℛ}_{ℯ\left(𝒾\right)}$ at the end of the round where the energy coefficient ξ is 0 <ξ≤1 in BASNs. Herein, the key objective function of the proposed scheme is to optimize the performance of each biosensor node by following the objectives defined in Equation (1) in each round of the simulation. Consequently, the objective function of the proposed scheme given in Eq.1 can be divided into single objectives, which can be written as

(6) $$\phi_{SARP}=\underset{\phi_{1}}{\underset{︸}{\int_{b_{\ell }}^{b_{𝓊}}\underset{\forall ℒ\left(𝒾\right)\in N}{\min}\;{ℒ}_{𝒾}\left(E_{𝒸}\right)}}+\underset{\phi_{2}}{\underset{︸}{\int_{b_{\ell }}^{b_{𝓊}}\underset{\forall ℒ\left(𝒾\right)\in N}{\min}\;{ℒ}_{𝒾}\left(D_{𝒾}\right)}}+\underset{\phi_{3}}{\underset{︸}{\int_{b_{\ell }}^{b_{𝓊}}\underset{\forall ℒ\left(𝒾\right)\in N}{\max}\;{ℒ}_{𝒾}\left(P_{𝒹𝓇}\right)}}+\underset{\phi_{4}}{\underset{︸}{\int_{b_{\ell }}^{b_{𝓊}}\underset{\forall ℒ\left(𝒾\right)\in N}{\max}\;{ℒ}_{𝒾}\left(T_{𝓅}\right)}}$$

In MILP, we define the following integer variables as

(7) ∀𝒾={1,2,…,𝓃}; ∀𝒿={1,2,…,𝓂}; ∀𝓀={1,2,…,ℓ};

(8a) $$X,Y=\{\begin{array}{cc} 1 & \mathrm{if}\;\mathrm{the}\;\mathrm{link}\;\mathrm{exists}\;\mathrm{between}\;S_{𝒾}\;\mathrm{and}\;S_{𝒿}\;\\ 0 & \mathrm{otherwise} \end{array},$$

(8b) $$A=\{\begin{array}{cc} 1 & \mathrm{if}\;\mathrm{the}\;\mathrm{sensor}\;\mathrm{node}\;S_{𝒾}\;\mathrm{is}\;\mathrm{active}\;\\ 0 & \mathrm{otherwise} \end{array},$$

(8c) $$ℛ=\{\begin{array}{cc} 1 & \mathrm{if}\;\mathrm{the}\;\mathrm{node}\;S_{𝒾}|S_{𝒽\left(𝒾\right)}\;\mathrm{cover}\;\mathrm{region}\;{ℛ}_{𝒾}\;\\ 0 & \mathrm{otherwise} \end{array};$$

(9a) $$\sum_{S_{𝒿}\in N_{𝓀}}X_{S_{𝒾},S_{𝒿}}-\sum_{S_{𝒿}\in N_{𝓀}}Y_{S_{𝓀},S_{𝒾}}={𝓉}_{f},\;\;S_{𝒾}\in V,$$

(9b) $$\sum_{S_{𝒾}\in S_{𝓃}}Y_{S_{𝓀},S_{𝒾}}\leq {𝓉}_{f}\left|S_{𝒾}\right|X_{S_{𝒿}},\;S_{𝒿}\in V_{S_{𝒾}\left(ℒ\left(𝒿\right)\right)},$$

(9c) $$\sum_{{ℊ}_{𝓀}}\sum_{S_{𝒿}\in {ℊ}_{𝓀}}{ℐ}_{S_{𝒿},S_{𝒽}}^{{ℊ}_{𝒾}}=S_{𝒽\left(𝓂𝒶𝓍\right)};$$

(10a) $${ℐ}_{S_{𝒿},S_{𝒽}}^{{ℊ}_{𝒾}}-{ℐ}_{S_{𝒿},S_{𝒽}}^{{ℊ}_{𝒾}}\leq 0,\;\forall \;1\leq S_{𝒽}\leq 𝒽\left(𝓂𝒶𝓍\right),\;S_{𝒿}\in {ℊ}_{𝒾},$$

(10b) $$S_{𝒽\left(𝒾\right)}\cdot {ℛ}_{𝒿}\geq 1,\forall 𝒾,𝒿;\;𝒿,\;\neq 𝒾,\;𝒿\neq 𝒾,1\leq \;𝒿\leq 𝓃,$$

(10c) $$\forall 𝒿=1,\ldots ,\left|𝓃\right|,\sum_{𝒾=1,\;𝒿\neq 𝒾}^{\left|𝓃\right|}C_{S_{𝒾},S_{𝒽𝒿}}\leq C_{𝓂𝒶𝓍},$$

(10d) $$\sum_{𝒿=1}^{𝓃}{ℐ}_{S_{𝒿},S_{𝒽}}^{{ℊ}_{𝒾}}=1,\;\forall ℊ,\;S_{𝒿}\in {ℊ}_{𝒾};$$

(11a) $$\forall S_{𝒽𝒾},\;𝒾=1,\ldots ,\left|𝓃\right|,\;{ℛ}_{𝒾}\leq A_{S_{𝒽𝒾}},$$

(11b) $$\sum_{𝒾=1}^{\left|𝓃\right|}{ℛ}_{𝒾}\cdot {𝒹}_{𝒾0}\left(S_{𝒽𝒾},S_{𝒽𝒿}\right)\geq 1\;\;𝒾\neq 𝒿;$$

(11c) $$\forall 𝒾=1,\ldots ,\left|𝓃\right|,\;\forall 𝒿=1,\ldots ,\left|𝓃\right|,𝒿\neq 𝒾,\;C_{S_{𝒽𝒾},S_{𝒽𝒿}}\leq A_{S_{𝒽𝒾}}\left({ℛ}_{𝒾}\right)-\;S_{𝒿};$$

(11d) $$\forall 𝒾=1,\ldots ,\left|𝓃\right|,\;\forall 𝒿=1,..,.\left|𝓃\right|,\;𝒿\neq 𝒾,\;C_{S_{𝒽𝒾},S_{𝒽𝒿}}\leq S_{𝒿},$$

(11e) $$\forall 𝒾=1,\ldots ,\left|𝓃\right|,\;\forall 𝒿=1,\ldots ,\left|𝓃\right|,\;𝒿\neq 𝒾,\;C_{S_{𝒽𝒾},S_{𝒽𝒿}}\leq 𝒹\;\left(S_{𝒽𝒾},S_{𝒽𝒿}\right),$$

(11f) $$\forall 𝒾=1,\ldots ,\left|𝓃\right|,\sum_{𝒿=1,\;𝒿\neq 𝒾}^{\left|𝓃\right|}C_{S_{𝒾}|S_{𝒽\left(𝒾\right)},S_{𝒽𝒿}}+S_{𝒿}=A_{S_{𝒾}|S_{𝒽𝒾}};$$

(12a) $$X_{S_{𝒿}}^{{ℊ}_{𝒾}}\geq Y_{S_{𝒽}}^{{ℊ}_{𝒾}}I_{S_{𝒿},S_{𝒽}}^{{ℊ}_{𝒾}},\;\forall \;1\leq S_{𝒿}\leq 𝓃,\;1\leq {ℊ}_{𝒾}\leq 𝓃,\;S_{𝒿}\in {ℊ}_{𝒾},$$

(12b) $$X_{S_{𝒿}}^{{ℊ}_{𝒾}}\geq Y_{S_{𝒽}}^{{ℊ}_{𝒾}}I_{S_{𝒽1},S_{𝒽2}}^{{ℊ}_{𝒾}},\;\forall \;1\leq S_{𝒿}\leq 𝓃,\;1\leq {ℊ}_{𝒾}\leq 𝓃,\;S_{𝒿}\in {ℊ}_{𝒾};$$

(13a) $${ℐ}_{S_{𝒽𝒾},S_{𝒽𝓀}}^{S_{𝒽𝒿}}\geq \left(S_{𝒽𝒿}/\sum_{𝓀}Z_{S_{𝒽\left(𝒿\right)}}^{S_{𝒽𝒾},S_{𝒽𝓀}}\right)\cdot {ℛ}_{𝓅\left(𝓀\right)}\;\forall \;𝒿,𝓀,𝒾;\;𝓀\neq 𝒿,\;\neq 𝒾,\;𝒿\neq 𝒾,$$

(13b) $${ℐ}_{S_{𝒽𝒾},S_{𝒽𝓀}}^{S_{𝒽𝒿}}\leq \left(S_{𝒽𝒿}/\sum_{𝓀}Z_{S_{𝒽\left(𝒿\right)}}^{S_{𝒽𝒾},S_{𝒽𝓀}}\right)\cdot {ℛ}_{𝓅\left(𝓀\right)}\;\;\forall \;𝒾,𝒿,𝓀;\;𝓀\neq 𝒿,\;\neq 𝒾,\;𝒿\neq 𝒾,$$

(13c) $$𝒹\left(S_{𝒽1},S_{𝒽2}\right)\leq {𝒹}_{𝓂𝒶𝓍}\;\;,\;S_{𝒽1}\in {ℊ}_{𝒾},\;S_{𝒽2}\in {ℊ}_{𝒿},$$

(13d) $${ℐ}_{S_{𝒽2}}^{{ℊ}_{𝒾}}\left(𝒹\right)\in S_{1},\;1\leq S_{𝒽}\leq 𝓃,1\leq {ℊ}_{𝒾}\leq 𝓃,S_{𝒽2}\in {ℊ}_{𝒿};$$

(14) $$\sum_{𝒾}^{𝓃}X_{{ℒ}_{1}\left(S_{𝒽1},S_{𝒽2}\right)}\left(DP_{𝒾}\right)\leq S_{𝒽2}\left({𝓈}_{𝒸}\right),\;S_{𝒽1}\in {ℊ}_{𝒾},\;S_{𝒽2}\in {ℊ}_{𝒿};$$

(15) $$\sum_{𝒾}^{𝓃}X_{{ℒ}_{𝓀}\left(S_{𝒽1},\;S_{𝒽2|𝒽3}\right)}\left(DP_{𝒾}\right),1\leq {ℒ}_{𝓀}\leq 𝓃,\;S_{𝒽1}\in {ℊ}_{𝒾},\;𝒽2|𝒽3\in {ℊ}_{𝒿};$$

(16) $$\sum_{S_{𝒿}\in N_{𝓀}}X_{S_{𝒾},S_{𝒿}}ℒ\left(𝒾\right)=\sum_{S_{𝒿}\in N_{𝓀}}Y_{S_{𝒾},S_{𝓀}}ℒ\left(𝒿\right)={𝓉}_{f},\;\;S_{𝒾}\in V;$$

(17a) $$E_{𝓉}\sum_{S_{𝒿}\in N_{𝓀}}X_{S_{𝒾},S_{𝒿}}+E_{𝓇}\sum_{S_{𝒿}\in N_{𝓀}}Y_{S_{𝓀},S_{𝒾}}\leq \;\xi {ℛ}_{ℯ\left(𝒾\right)},\;\;S_{𝒾}\in V,$$

(17b) $$E_{𝓉}\sum_{S_{𝒿}\in N_{𝓀}}X_{S_{𝒾},S_{𝒿}}+E_{𝓇}\sum_{S_{𝒿}\in N_{𝓀}}Y_{S_{𝓀},S_{𝒾}}\leq \;E_{𝓂𝒶𝓍},\;\;S_{𝒾}\in V;$$

(18a) $$\forall 𝒾=1,\ldots ,\left|𝓃\right|,\;\forall \;𝒿=1,\ldots ,\left|𝓃\right|,\;𝒿\neq 𝒾,\;C_{S_{𝒾},S_{𝒽𝒿}}\leq C_{S_{𝒽𝒿}};$$

(18b) $$\forall 𝒾=1,\ldots ,\left|𝓃\right|,\;\forall \;𝒿=1,\ldots ,\left|𝓃\right|,\;𝒿\neq 𝒾,\;C_{S_{𝒽𝒿},S_{𝒾}}\leq C_{S_{𝒽𝒿}},$$

(18c) $$\forall 𝒾=1,\ldots ,\left|𝓃\right|,\;\forall \;𝒿=1,\ldots ,\left|𝓃\right|,\;𝒿\neq 𝒾,\;C_{S_{𝒾}|S_{𝒽\left(𝒾\right)},S_{𝒽𝒿}}\leq 𝒹\;\left(S_{𝒽𝒾},S_{𝒽𝒿}\right){ℛ}_{𝓅\left(𝓀\right)};$$

(19) $$\forall ℛ=1,\ldots ,\left|{ℛ}_{𝓃}\right|,\sum_{𝒾=1}^{\left|𝓃\right|}A_{S_{𝒾}|S_{𝒽𝒾}}\cdot {ℛ}_{𝒾}\geq 1.$$

Constraints in (9a) show the flow of messages at each biosensor node in the network. In (9b), constraints ensure that the transmission of messages to the neighboring nodes is possible only if a biosensor node exists in that region. In (9c), constraints verify that the sum of current group head nodes $S_{𝒽}$ is not more than the maximum defined head nodes $S_{𝒽\left(𝓂𝒶𝓍\right)}$. Constraints in (10a) guarantee that a biosensor node $S_{𝒿}$ in a group ${ℊ}_{𝓀}$ uses a biosensor node as a group head node $S_{𝒽}$ only if it has been chosen to act as a cluster head. In (10b), constraints show that more than one cluster head exists in the region ${ℛ}_{𝒿}$. Constraints in (10c) define the upper bound of each cluster size. Constraints in (10d) guarantee that each biosensor node in a group is linked to the group leader, where the ${ℐ}_{S_{𝒾},S_{𝒿}}^{{ℊ}_{𝓀}}$ is a binary variable, which indicates that a sensor node $S_{𝒿}$ in a group ${ℊ}_{𝓀}$ uses a group leader sensor node $S_{𝒽}$ as a master information aggregator. Constraints in (11a)–(11f) show that each active biosensor node is connected to one group leader or a group leader connected to at least one other group leader within its transmission range in the network, in which $𝒹\;\left(S_{𝒽𝒾},S_{𝒽𝒿}\right)$ is equal to 1 only if the group leader $S_{𝒽𝒾}$ can directly reach $S_{𝒽𝒿}$, and is ${𝒹}_{𝒾0}\left(S_{𝒽𝒾},S_{𝒽𝒿}\right)$ otherwise. C is an integer whose value is 1 when a biosensor node is connected to the cluster head $\left(C_{S_{𝒾},S_{𝒽𝒿}}\right)$ or a cluster leader is connected to the neighboring group leader $\left(C_{S_{𝒽\left(𝒾\right)},S_{𝒽𝒿}}\right)$, and 0 otherwise. Constraints in (12a) state that all the packets are collected by the group head node from its member nodes in a cluster. Constraints in (12b) ensure that the collected data are forwarded from the biosensor head node $S_{𝒽1}$ to the neighboring biosensor head node $S_{𝒽2}$. Constraints in (13a) and (13b) state that a relay cluster head node joins the routing path ${ℛ}_{𝓅\left(𝓀\right)}$ between the source and the destination in the network, in which ${ℐ}_{S_{𝒽𝒾},S_{𝒽𝓀}}^{S_{𝒽𝒿}}$ is a binary variable whose value is 1 only if a group leader biosensor node $S_{𝒽𝒿}$ is on the route between the source head node $S_{𝒽𝒾}$ and the destination head node $S_{𝒽𝓀}$, while $Z_{S_{𝒽\left(𝒿\right)}}^{S_{𝒽𝒾},S_{𝒽𝓀}}$ is also a binary indicator that is equal to 1 only if the traffic stream, sourced at $S_{𝒽𝒾}$, and the destination to $S_{𝒽𝓀}$ uses the link to the head $S_{𝒽𝒿}$ node in the network. In (13c), constraints guarantee that the distance between two group leaders must not be greater than the defined maximum distance ${𝒹}_{𝓂𝒶𝓍}$. Constraints in (13d) ensure that the group head node $S_{𝒽2}$ is closer to the sink. Constraints in (14) declare that a group head node $S_{𝒽2}$ can not receive the data packets $DP_{𝒾}$ from the neighboring group leader $S_{𝒽1}$ over a link ${ℒ}_{1}$ than its maximum storage capacity ${𝓈}_{𝒸}$. This constraint helps to avoid data packet losses due to buffer overflow during packet transmission in the network. Constraints in (15) support the constraints of (14), which guarantee that the data packets always move over a different link ${ℒ}_{𝓀}$ towards the neighboring cluster head nodes in the network. Constraints in (16) ensure that the number of packets transmitted by a sensor node $S_{𝒾}$ to sensor node $S_{𝒿}$ over a link ℒ(𝒾) is equal to the number of packets transmitted from the sensor node $S_{𝓀}$ over a link ℒ(𝒿). This helps to balance the network traffic load in the entire network. Constraints in (17a) state that the sum of the energy spent by the biosensors during packet transmission $\left(E_{𝓉}\right)$ and reception $\left(E_{𝓇}\right)$ is bounded to a specific round in the network. Constraints in (17b) minimize packet transmission and reception energy consumption of each biosensor node in each round of the data collection process. Constraints in (18a)–(18c) state that the routing constraints show that the biosensor nodes and head relay nodes along a routing path ${ℛ}_{𝓅\left(𝓀\right)}$ between the source and the destination are connected in the network. Constraints in (19) make it clear that at least one group leader or a sensor node in the region ${ℛ}_{𝒾}$ exists away from the sink in the network.

## 5. Path Loss and Energy Consumption Models

In BASNs, the signals transmitted by biosensors may diffract around the body or reflect a nearby distraction and then back at the body. Therefore, the link quality cannot be described by a simple power–distance law since each link between biosensors faces different fading, shadowing, polarization, interference, and correlation parameters in BASNs [40]. Considering these factors, the path loss (PL) model [41] over the distance (𝒹) between biosensor nodes $S_{𝒾}$ and $S_{𝒿}$ can be indicated as

(20a) $$PL\left(𝒹\right)=PL\left({𝒹}_{0}\right)+10𝓃\ell ℴℊ\left(\frac{𝒹}{{𝒹}_{0}}\right).$$

The average path loss of the whole body can be computed as

(20b) $$PL\left({𝒹}_{0}\right)=10*\ell ℴℊ\left(4\pi \cdot {𝒹}_{0}\left(f\right)\right)*S_{\ell }\;.$$

The total path loss by considering the shadowing factor can be numerically computed as

(20c) $$PL=PL\left(𝒹\right)+X_{\sigma }.$$

In the energy consumption model [42], the energy required to transmit an ℓ bit data message between two nodes separated by distance 𝒹 can be calculated as

(21a) $${Ε}_{𝓉𝓍}\left(𝒹,\ell \right)={Ε}_{𝓉𝓍}\left(\ell \;\right)+{Ε}_{𝒶𝓂𝓅}\left(𝒹\right)*\ell \;.$$

The energy required by the transmit amplifier, with the distance $𝒹\leq {𝒹}_{𝓉𝒽}$ and $𝒹>{𝒹}_{𝓉𝒽}$, to maintain the lowest signal-to-noise ratio for effective wireless channel transmission is given by

(21b) $${Ε}_{𝒶𝓂𝓅}\left(𝒹\right)=\{\begin{array}{c} {Ε}_{𝒻𝓈}*{𝒹}^{2},\;𝒹\leq {𝒹}_{𝓉𝒽}\;\\ {Ε}_{𝒻𝓈}*{𝒹}^{4},\;𝒹>{𝒹}_{𝓉𝒽}\; \end{array},$$

where

(21c) $${𝒹}_{𝓉𝒽}=\sqrt{\frac{{Ε}_{𝒻𝓈}}{{Ε}_{𝒶𝓂𝓅}}}\;.$$

Consequently, the energy needed for receiving a bit (ℓ) at the given reference distance (𝒹) can be calculated as

(21d) $${Ε}_{𝓇𝓍}\left(\ell \right)={Ε}_{\mathrm{elec}}\left(\ell \right),$$

in which PL (${𝒹}_{0}$), *f*, $S_{\ell }$, $E_{ℯ\ell ℯ𝒸}$, $E_{𝒻𝓈}$, ${Ε}_{𝒶𝓂𝓅}$, and ${𝒹}_{𝓉𝒽}$ are, respectively, the path loss in dB at a reference distance, the frequency of operation, the speed of light, the transmitter energy or receiving circuit, the parameters in a free space model, the parameters in a multipath fading model, and the threshold in meters. During simulation studies, two other routing schemes, namely Energy Aware Link Efficient Routing Approach ELR-W [21] and Clustering Routing Protocol for Boday Area Sensor Network (CRPBA) [23] are used as comparisons to evaluate the performance of our proposed protocol in BASNs. The simulation parameters are given in Table 3.

## 6. Performance Analysis

**Figure 2:**
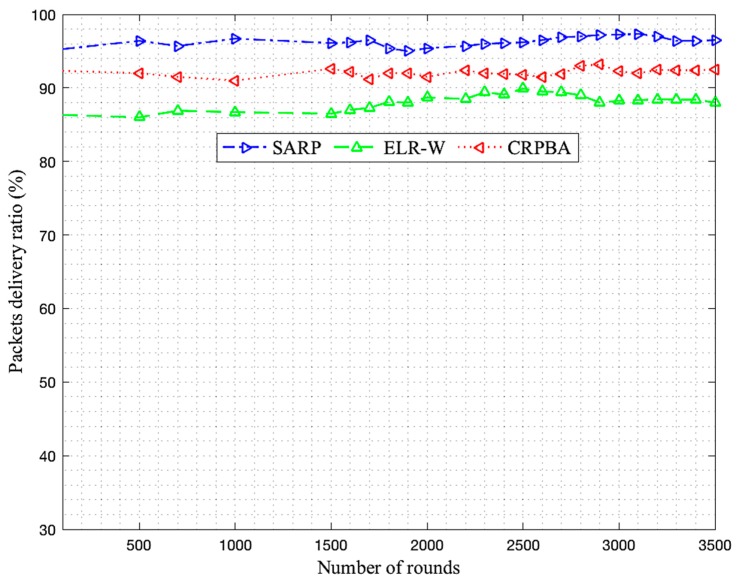
Packet delivery ratio vs number of rounds.

The body area sensor network collects data from the biosensors located on the patient’s body, which may in certain cases be critical. Therefore, the developed scheme must provide data to the medical server or physician located at a remote location for on-time analysis. This will only be feasible if the collector gathers information from the biosensors in time by considering various data reliability requirements of both emergency and nonemergency data. In this respect, reliable data delivery is one of the key metrics, which depends on various other metrics such as packet error rate, throughput, and latency. These factors determine the competency of a routing protocol against the others, especially for a limited energy body area sensor network in order to increase lifetime. Consequently, the packet reception rate for each SARP, ELR-W, and CRPBA routing scheme is illustrated in Figure 2. In the proposed scheme, the entire packet delivery ratio is divided into low, medium, and high levels. The high level is considered as the best data gathering performance level in the network. Initially, around 500 rounds, the packet reception rate of SARP protocol was recorded as high, up to 95%, compared to ELR-W and CRPBA protocols, which were observed to be low, around 91.1% and 86.9%, respectively. However, as time passed and the number of rounds increased between 2200 to 2400, the data delivery rate performance of SARP significantly improved and carried more data packets around 96.7% in BASNs. In contrast, the data delivery performances of both ELR-W and CRPBA protocols were observed at a medium level, around 91.6% and 89%, respectively. Finally, the packet delivery ratios of SARP, ELR-W, and CRPBA routing protocols were recorded the highest at around 98.4%, 92.3%, and 86.9% at round numbers between 3000 and 3500. All these facts indicate that the SARP routing protocol performed the best in achieving high packet delivery rates compared to all other schemes in BASNs. 

**Figure 3:**
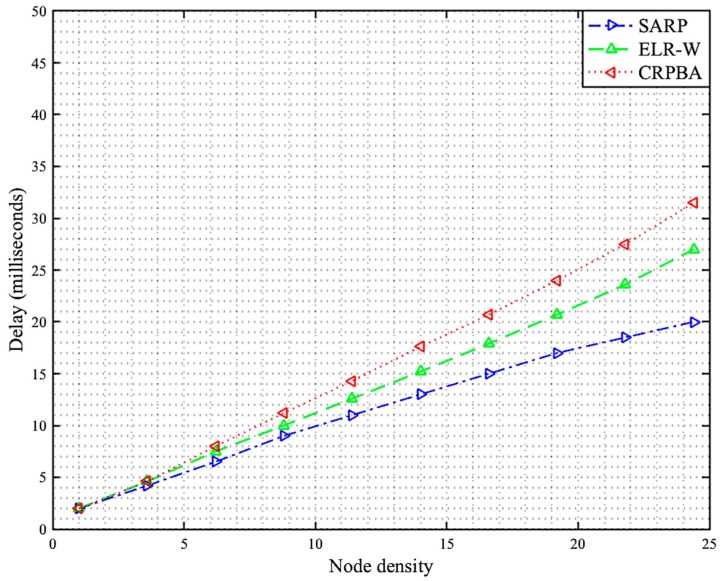
Latency vs node density.

Generally, the time lag between the sender biosensor and data collector receiver is referred to as delay, and it is a very important factor in the BASNs. The delay values of SARP, ELR-W, and CRPBA protocols are depicted in Figure 3. In the proposed scheme, the latency values are divided into low, medium, and high in the network. In the beginning, the latency value of SARP protocol was observed to be low, around 10 ms, when up to 8 sensors were involved in the packet forwarding process. In contrast, the latency values of ELR-W and CRPBA protocols were observed up to 10 and 11.3 ms with similar node densities. However, as time passed, the latency values of SARP, ELR-W, and CRPBA protocols increased to a medium level, up to 12, 15, and 17 ms, when up to 15 sensors were involved in forwarding patient data in the network. The latency values of SARP, ELR-W, and CRPBA protocols further increased to high levels, up to 17, 20.8, and 24 ms, when up to 20 sensors were involved in forwarding patient data in the network. The highest latency value for SARP was recorded around 20 ms, when the node density was up to 25. In contrast, the latency values of both ELR-W and CRPBA protocols with similar node densities were observed around 27 and 32 ms, respectively. These simulation facts show that the SARP scheme performed the best in achieving low latency values for low- to high-density networks. This best performance of SARP was due to its packet transmissions on dedicated paths, which were established by considering the link quality between relay biosensors towards the sink. In addition, the clustering architecture reduced the burden on forwarders since each cluster head gathered data from its members and fused it before sending. Thus, more data packets were transmitted over the links between the forwarders in a robust and reliable manner compared to all other schemes. In SARP, the transmission of a patient’s emergency data over several nodes is avoided in the network. In case of a route failure, a packet forwarder considered an alternative node as a potential forwarder towards the sink without conflicting situations. Thus, a significant amount of time spent in negotiation with the neighboring nodes is saved in the packet forwarding process. Because of these reasons, the developed protocol performed better in terms of minimizing overall network latency. 

The main issue of high latency in the ELR-W scheme is that more initial calculations are required to check various packet forwarding conditions before data transmission, which consumes more processing time at each relay node along a routing path and, thus, leads to more delay. However, the latency of packet forwarding gradually decreased with time once the initial calculations were done; therefore, it performed better than CRPBA protocol. In addition, direct communication over a longer distance also helps ELR-W to convey packets in a robust manner towards the sink. However, this long-distance communication with lower link quality creates a number of corrupted data packets in ELR-W protocol. On the other hand, one of the main reasons for the high latency in CRPBA is its unstable cluster architecture, which contains small-sized and large-sized clusters. Therefore, the average number of hops visited by the data packets increases between the source and the sink, which contributes to latency. This also increases the probability of data path loops; thus, the data packets do not reach the destination at a specific time. In addition, the data path loop also causes congestion for the relay nodes along a routing path since nodes keep transmitting the packets, which impact the communication delay. In addition, the CRPBA protocol has no specific policy to forward the packets on alternate paths in case of a route node failure. In the route recovery process, it spends a significant amount of time in negotiation with the neighboring nodes in order to find a new forwarder node. Therefore, in most cases, the sensitive data become invalid due to lack of an available path towards the sink. These invalid data packets reduced the overall packet delivery ratio in the CRPBA scheme.

**Figure 4:**
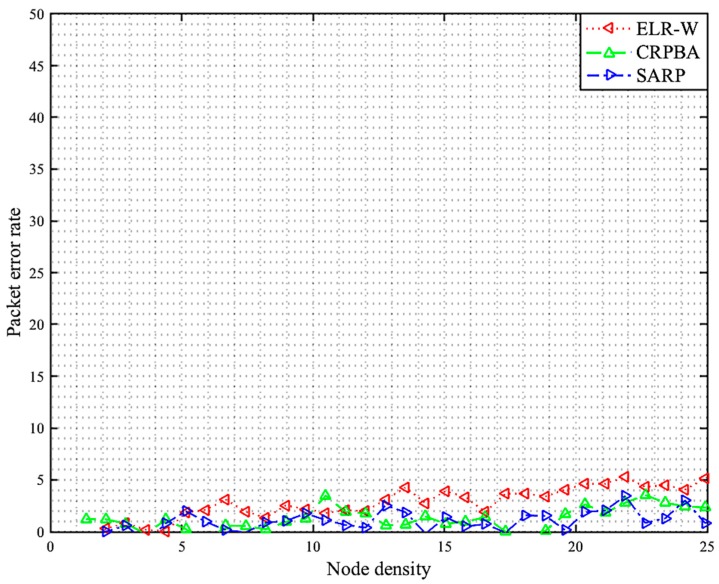
Packet error rate vs node density.

**Figure 5:**
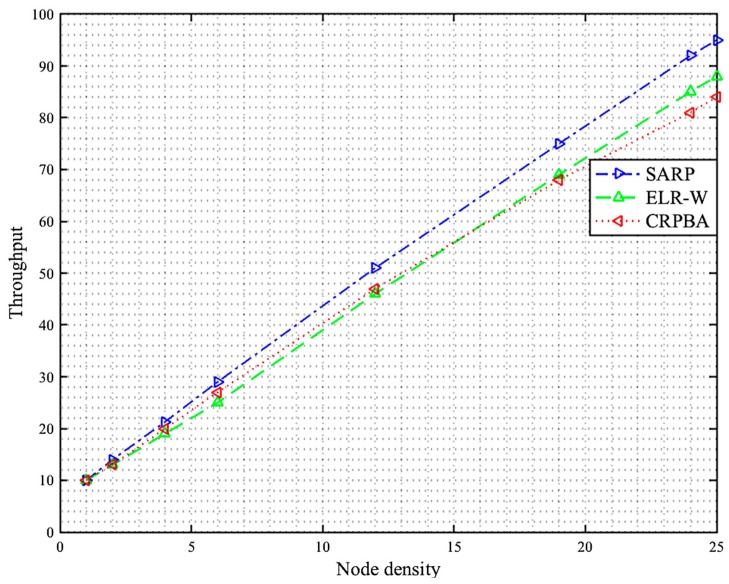
Throughput vs node density.

However, the small size clustering architecture reduces the burden on relay nodes even when a huge amount of patient data is routed in the network. Therefore, the low packet error rate performance of CRPBA is fairly conclusive compared to ELR-W, as shown in Figure 4. The main disadvantage of the ELR-W routing protocol, which results in a high packet error rate, is its lack of considering the interference issues during link stability measurements between the forwarder nodes. In addition, it does not always select the shortest path during packets forwarding, which not only increases the chance of packet loss due to path loops but also creates excessive interference for neighboring nodes in the network. The SARP protocol compared to CRPBA and ELR-W protocols achieved the lowest packets error rate, around 2.25% of the originated data packets, as shown in Figure 4. This best performance of a low packet error rate in SARP is due to its high link quality between sensors and cluster leaders and forwarder cluster heads. In addition, the designed scheme during the packet forwarding process appropriately considers the hop counts, which further reduces the probability of corrupted packets occurring in BASNs. All aforesaid factors result in a high network throughput in SARP compared to CRPBA and ELR-W protocols, as shown in Figure 5. In fact, throughput is the measurement of the number of packet transfers over a link in a given amount of time. It is usually computed in bits per second (bps). Simulation facts, shown in Figure 5, make it clear that SARP achieved more than 96% throughput and outperformed the ELR-W than CRPBA protocols in BASNs. Comparatively, the better throughput performance of ELR-W than CRPBA is because it forwards all types of data on a priority basis without blocking the transmission of other biosensors in a life-critical situation of the patient.

**Figure 6:**
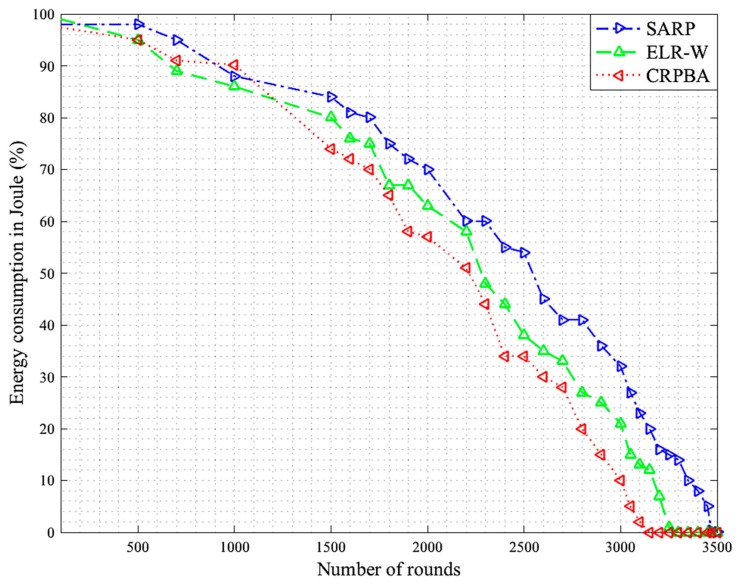
Residual energy vs number of rounds.

The energy consumption of a node is defined as the sum of energy consumed in various activities, such as observing vital signs and receiving and transmitting packets in BASNs. The continuous packet transmission of vital signs consumes a high level of energy for the biosensors and, therefore, must be minimized in order to increase the lifespan of BASNs. Figure 6 illustrates that, in terms of low energy consumption, SARP outperformed the CRPBA and ELR protocols and completed almost 3500 rounds with different data traffic loads in BASNs. With more details, in initial rounds around 500, the residual energy profile of the SARP protocol was recorded around 97.4%, compared to ELR-W and CRPBA protocols, which were observed up to 96.8% and 96.6%, respectively. However, as time passed and the number of rounds increased between 2000 and 2300, the low-energy consumption performance of SARP significantly improved and recorded around 59.7% in BASNs. In contrast, the low-energy consumption profiles of both ELR-W and CRPBA protocols were observed around 55.7% and 55.1%, respectively.

The low-energy consumption profile of ELR-W was found to be significantly better than the CRPBA protocol in WBASNs. Finally, the energy consumption profiles of SARP, ELR-W, and CRPBA protocols were recorded around 1.85%, 0.5%, and 0.3% for round numbers of 3500, 3300, and 3200, respectively. SARP achieved the best low-energy consumption profile because it exchanged the lowest number of control packets in the network. SARP periodically maintained and updated its neighbors and routing tables by exploiting the data packets; thus, no extra control packets are required, and data paths are always up to date. In addition, during a route recovery process, it used the least amount of control message overhead to search for an appropriate next-hop packet forwarder, which significantly saved node energy. Moreover, by assigning alternate paths, it significantly reduced the hotspot problems and balanced the traffic load and residual energy of the cluster heads in BASNs. On the other hand, the low-energy consumption profile of CRPBA was found to be much higher than ELR-W, but its performance gradually declined when more biosensors transmitted packets in increasing numbers of rounds. The main problem of high energy consumption in CRPBA is to re-establish the routes and re-transmit the dropped/delayed packets. Therefore, because of an increased number of packet re-transmissions, the nodes in CRPBA were not able to go into sleep mode in order to save energy. On the contrary, the main issue of high energy consumption in ELR-W is that control message overheads occurred when the data packets did not reach the destination, because of relay note failure, but the relay nodes re-transmitted the packets and kept updating the routes in BASNs. Thus, it consumed a notable sum of node energy; however, it performed better than the CRPBA routing protocol in BASNs. 

In sum, compared to all other schemes, the proposed scheme provides an energy-efficient and quality-aware delivery of emergency data to the physician in a life-critical situation of the patient.

## 7. Conclusions

Biosensors face severe issues, such as unreliable data transmission and short life spans, because of poor communication efficiency caused by the complexity of the body tissues and unpredictable body movements in BASNs. Therefore, reliable and sustainable transmission of the instant data collected from these body sensors to the observers (e.g., doctors, nurses, etc.) is extremely challenging, which results in a poor monitoring picture of the health condition of the patients. Thus, a more advanced data transmission mechanism is greatly needed to optimize resource utilization and to overcome network reliability and instability issues for BASN-based health monitoring applications. To this end, this research proposed a novel, multiobjective routing protocol for BASNs-based IoT healthcare applications. The proposed scheme, by employing a novel, multiobjective lion optimization mating architecture, reduced local search problems when finding the best routing paths between the source and the destination in BASNs. Extensive simulation studies were performed through a network simulation tool, namely MATLAB 9.5 (R2018b), to validate the performance of the proposed scheme against the existing routing protocols. The study results show that the SARP scheme performed best in terms of the high packet delivery ratio with low latency, packet error rates, and network energy consumption, at the expense of data redundancy, for BASN-based health monitoring applications. As future work, research may focus on a security-aware routing mechanism in BASN-based health monitoring applications.

## Figures and Tables

**Table 1:** Comparison of schemes in BASNs.

Sr. No.	Routing Protocols	Static- Channel	Architecture	Packet Delivery Ratio	Delay	Energy Consumption	Packet Error Rate	Throughput	Reliability	Robustness	Convergence
1	Co-LAEEBA [16]	✓	Flat	✓		✓		✓			
2	QPRD [17]	✓	Flat	✓	✓	✓					
3	M-ATTEMPT [18]	✓	Flat	✓		✓		✓			
4	E-OCER [25]	✓	Flat	✓		✓					
5	ORACE-Net [19]	✓	Flat	✓	✓	✓					
6	TTRP [20]	✓	Flat	✓	✓	✓		✓			
7	OEABC [26]	✓	Flat	✓		✓					
8	CRPBA [23]	✓	Clustering			✓					
9	REPC [12]	✓	Flat	✓		✓					
10	ELR-W [21]	✓	Flat	✓		✓					
11	DSCB [24]	✓	Clustering	✓		✓		✓			
12	Tripe-EEC [2]	✓	Flat	✓	✓	✓					
13	ATAR [22]	✓	Flat	✓		✓		✓			
14	SARP (Proposed)	✓	Clustering	✓	✓	✓	✓	✓	✓	✓	✓

**Table 2:** Notations used in SARP.

Notation	Explanation
${ℒ}_{𝒾}\left(E_{𝒸}\right)$	$E_{𝒸}$ is the energy consumption subject to minimization by lion ${ℒ}_{𝒾}$.
${ℒ}_{𝒾}\left(D_{ℯ}\right)$	$D_{ℯ}$ is the delay needing to be minimized by lion ${ℒ}_{𝒾}$.
$L_{𝒾}\left(P_{𝒹𝓇}\right)$	$P_{𝒹𝓇}$ is the packet delivery ratio needing to be increased by lion ${ℒ}_{𝒾}$.
${ℒ}_{𝒾}\left(T_{𝓅}\right)$	$T_{𝓅}$ is the throughput subject to being increased by lion ${ℒ}_{𝒾}$.
$b_{\ell }$,$b_{𝓊}$	indicates the lower and upper bounds of the search space, respectively.
$ℳ{\left(ℋ\right)}_{𝒾}$	represents the matrix for saving the position of each hunter by considering the $P_{𝒾\left(\ell \right)}$ position.
${ℋ}_{𝒾,𝒿}$	is the value of the 𝒿th dimension of the 𝒾th hunter.
𝓃	is the number of hunters, and 𝒹 is the number of variables.
$P_{𝒾\left(\ell \right)}$	is the current location (ℓ) of the prey.
$P_{𝒾\left(\ell \right)}\left(𝓃ℯ𝓌\right)$	is the new position of the prey.
${ℋ}_{𝒿\left(\ell \right)}$	is the current position of the hunter.
δ	is the percentage of improvement in the fitness of the hunter.
${ℋ}_{𝒿}\left(𝓃ℯ𝓌\right)$	is the network position of the hunter.
𝓇(0,1)	is a random number whose value is either 0 or 1.
𝒹	is the distance between two lions or between lions and prey in the search space.
${𝒹}_{1},{𝒹}_{2}$	is the distance between the female lion’s location and the certain point chosen by tournament selection among the pride’s territory.
${𝓋}_{1},{𝓋}_{2}:{𝓋}_{1}$	is a vector whose start point is the previous location of the $ℱ{ℒ}_{𝒾}$ and its direction is toward the selected position $ℱ{ℒ}_{𝒾}\left(𝓃ℯ𝓌\right)$, and ${𝓋}_{2}$ is perpendicular to ${𝓋}_{1}$, i.e., ${𝓋}_{1}\times {𝓋}_{2}=0$ and ${𝓋}_{1}=1$.
${𝓇}_{1},{𝓇}_{2}$	are random vectors with values in [0, 1].
$\vec{\alpha }$	linearly decreases from 2 to 0 over the course of iterations.
$\vec{ℛ}\;\in {\vec{ℛ}}_{1},{\vec{ℛ}}_{2}$	is a vector that generates random values greater than 1 or less than −1.
$\vec{C}\in {\vec{C}}_{1},{\vec{C}}_{2}$	is a vector that generates random values in [0, 2].
$S\left({ℋ}_{𝒿\left(\ell \right)}\right)$	is the number of lions in a pride $𝒾\in \;G_{𝒾}\left(ℛ{ℒ}_{\left(𝒿\right)}\right)$, which improves their fitness in the last iteration.
${ℋ}_{𝒽\left(𝒾\right)}^{𝒿}$	shows the position of the selected 𝒾th hunter at the 𝒿th iteration.
ℓ	is the position of the prey or a hunter in the search space.
${𝒹}_{\left(ℳ{ℒ}_{𝒾},ℳ{ℒ}_{𝒿}\right)}$	is the distance between the male lion’s position and the selected area of territory.
θ	is the angle to search for a wider area around the current solution in the search space.
𝓊	is a random, uniformly distributed number between −π/6 and π/6.
β	is a randomly generated number with a normal distribution with mean value 0.5 and standard deviation between 0 and 1.
*X*σ	is a shadowing factor in dB, which is a Gaussian-distributed random variable with mean zero and standard deviation σ.
$E_{𝒶𝓂𝓅}$ (*r*)	is the energy required by the transmit amplifier to maintain an acceptable signal-to-noise ratio to transfer data messages reliably.
𝓃	is the path loss exponent, considered as 2 in free space, and varies for different body locations.

**Table 3:** Values of parameters used in SARP.

Parameters	Value (s)
Channel	Body channel
Network topology	Deterministic
Biosensor deployment area	2×3 $m^{2}$
Sink location area	30×50 ${\mathrm{cm}}^{2}$
Initial node energy	0.5 J
Initial sink energy	10 kJ
Number of biosensor nodes	25
Number of sink nodes	1
Number of female lions	10
Number of male lions	14
Cost of high transmission	30 nJ/bit
Cost of low transmission	23 nJ/bit
Cost of reception	7 nJ/bit
Idle power	0.90 nJ
Data aggregation power	5 nJ/bit/signal
Signal amplifying power	10 pJ/bit/$m^{2}$
High communication range of sensors	0.5 m
Low communication range of sensors	0.3 m
Transmission range of sink	1 m
Line-of-sight (LOS)	3.38
Non-line-of-sight (NLOS)	5.90
Bandwidth	20 MHz
Maximum data rate	151.8 kbps
Packet size	3 kb
Control packet size	50 bits
Packet generation rate	0.01~0.1 packets/s
Memory size	0.3 MB
Modulation scheme	DPSK
Physical layer	IEEE 802.15.6
Antenna	Omnidirectional
Simulation time per epoch	80 s
Number of runs	53

## Supplementary Materials

Supplementary File 1
